# The evolutionary history of the polyQ tract in huntingtin sheds light on its functional pro-neural activities

**DOI:** 10.1038/s41418-021-00914-9

**Published:** 2022-01-01

**Authors:** Raffaele Iennaco, Giulio Formenti, Camilla Trovesi, Riccardo Lorenzo Rossi, Chiara Zuccato, Tiziana Lischetti, Vittoria Dickinson Bocchi, Andrea Scolz, Cristina Martínez-Labarga, Olga Rickards, Michela Pacifico, Angelica Crottini, Anders Pape Møller, Richard Zhenghuan Chen, Thomas Francis Vogt, Giulio Pavesi, David Stephen Horner, Nicola Saino, Elena Cattaneo

**Affiliations:** 1 Department of Biosciences, University of Milan, 20122 Milan, Italy; 2 Istituto Nazionale Genetica Molecolare, Romeo ed Enrica Invernizzi, 20122 Milan, Italy; 3 Department of Biology, University of Roma Tor Vergata, 00173 Rome, Italy; 4 CIBIO, Research Centre in Biodiversity and Genetic Resources, InBIO, Universidade do Porto, 4485-661 Vairão, Portugal; 5 Ecologie Systématique Evolution, Université Paris-Sud, CNRS, Agro ParisTech, Université Paris-Saclay, 91405 Orsay, CEDEX France; 6 CHDI Management/CHDI Foundation, Princeton, NJ 08540 USA; 7 Department of Environmental Science and Policy, University of Milan, 20133 Milan, Italy

**Keywords:** Cell biology, Genetics, Neuroscience, Neurological disorders

## Abstract

Huntington’s disease is caused by a pathologically long (>35) CAG repeat located in the first exon of the Huntingtin gene (*HTT*). While pathologically expanded CAG repeats are the focus of extensive investigations, non-pathogenic CAG tracts in protein-coding genes are less well characterized. Here, we investigated the function and evolution of the physiological CAG tract in the *HTT* gene. We show that the poly-glutamine (polyQ) tract encoded by CAGs in the huntingtin protein (HTT) is under purifying selection and subjected to stronger selective pressures than CAG-encoded polyQ tracts in other proteins. For natural selection to operate, the polyQ must perform a function. By combining genome-edited mouse embryonic stem cells and cell assays, we show that small variations in HTT polyQ lengths significantly correlate with cells’ neurogenic potential and with changes in the gene transcription network governing neuronal function. We conclude that during evolution natural selection promotes the conservation and purity of the CAG-encoded polyQ tract and that small increases in its physiological length influence neural functions of HTT. We propose that these changes in HTT polyQ length contribute to evolutionary fitness including potentially to the development of a more complex nervous system.

## Introduction

Huntington’s disease (HD) is a neurological genetic disorder caused by an anomalous expansion of a >35 CAG triplet repeats in the huntingtin gene (*HTT*) that leads to an abnormally elongated polyglutamine (polyQ) tract [1–3]. It is currently unknown whether HD pathogenesis occurs at the DNA, RNA, or protein levels [4–7]. Within the non-pathogenic range of CAGs, the *HTT* gene encodes for a protein (HTT) which is implicated in multiple fundamental physiological processes. Among these functions, HTT regulates neurogenesis and brain development, together with the survival and function of cortical and striatal neurons in the adult brain [8, 9]. Molecularly, HTT scaffolds a large repertoire of binding proteins to coordinate several cellular processes, including gene transcription, synaptic activity, vesicles trafficking and autophagy [8–10]. In spite of this knowledge, the contribution of the non-pathogenic CAG tract itself and its highly polymorphic lengths in normal *HTT* function remains poorly examined.

In the general human population, the non-pathogenic CAGs number ranges from 9 to 35. More remarkably, its distribution is right-skewed [11–13] with a higher frequency of longer CAG tracts in the highest normal CAGs range between 27–35 [12], thus suggesting that in humans there is a bias towards longer alleles transmission. This unexpected finding, together with the evidence that CAGs have arisen and have been retained throughout evolution suggest that CAG encoding repeats may have a positive role in adaptive evolution and might exert a function [14–19].

Earlier hypothesis proposed that the CAG repeat tracts have little or no effect on the normal function of the gene in which they are located [20, 21]. In contrast, initial studies in humans reveal that longer non-pathogenic CAG size in *HTT* gene are associated with a phenotype. In particular, CAG repeats below disease threshold were described to have an impact on brain development and general intelligence [22]. Furthermore, the size of the non-pathogenic trinucleotide repeats has been found to affect normal brain structure in humans [23], and more CAGs in the non-pathogenic range were suggested to have an impact on cognition [24, 25]. Together, these data indicate that the non-pathogenic CAG tract in *HTT* may confer advantageous effects to the DNA, RNA and/or protein. Moreover, our earlier work speculated that CAGs may have been subjected to selective pressure during evolution because greater number of repeats are found in species sharing a more recent common ancestor with humans. We reported that Echinodermata and Cephalochordata are the first groups of organisms to carry a polyQ (only 2Q) tract in the HTT protein [17, 26], and curiously are among the most basal lineages to develop a primitive nervous system [27, 28]. Vertebrates, instead, have four or more Qs, with the highest number found in humans [17]. These results again support the hypothesis that the CAG sequence, which has survived and has been preserved to this day, has a fundamental role and a defined purpose.

Here we directly tested the hypothesis that the length and purity of the non-pathogenic CAG tract in *HTT* have been promoted by natural selection and that the tract length is functionally active. First, by studying the conservation of the CAG tract from over 200 species, the largest sequencing effort of *HTT* exon1 to date, we provide evidence that the HTT polyQ tract is under purifying selection and subjected to stronger selective pressures than any other polyQ-containing protein. Second, by using genome-edited mouse embryonic stem (mES) cells that carry a stepwise increase of CAGs in the normal *HTT* gene, we show that the polyQ tract itself is functionally relevant. Importantly, small increases in the non-pathogenic CAG length positively and significantly correlate with the cells’ neurogenic potential and with transcriptional alterations in pathways that control general neuronal organization and function/s. Taken together, these results demonstrate that natural selection acts to preserve an uninterrupted contiguous Q stretch in HTT and that its progressive increase in length gradually enhances the biological, pro-neurogenic activity of the normal protein as we demonstrate by stem cell based assays.

## Materials and methods

### Sequence retrieval from databases

Sequences were obtained from four online resources: NCBI (www.ncbi.nlm.nih.gov), UCSC (www.genome.ucsc.edu), Ensembl (www.ensembl.org) and UNIPROT (www.uniprot.org). Coding sequences were obtained as nucleotide mRNA predictions either directly or as GenBank files. BLAST searches using Human *HTT* (Hg18) full-length exon1 sequence were performed in unannotated genomic assemblies. Specific SRA (Sequence Read Archive) experiments were also searched for *HTT* orthologous genes using sequences from relatively close species as queries.

### Sample collection and extraction of genomic DNA

gDNA extraction was performed either using a slightly modified Phenol/Chloroform protocol or using a commercial kit (NucleoSpin Tissue® kit—Machery-Nagel, REF 740952). Quality and concentration of all extracted DNA samples were verified on a gel and by spectrometer analysis (NanoDrop 1000—ThermoScientific).

### Amplification of HTT exon1 orthologs

A general PCR protocol was developed to assess the nucleotide sequence of exon1 in the *HTT* orthologous genes. Primers were designed in the most conserved outer regions of *HTT* exon1 to span the entire repeat region and to produce amplicons between 100–300 bp, suitable for direct sequencing or cloning (see Supplementary dataset S22 for the primer list). A top-quality replication enzyme, the Platinum Taq DNA Polymerase High Fidelity (kit Life Technologies, CAT n°11304–011) was used for PCR amplification. PCR reactions were assembled under a sterile fume hood and working environment was cleaned with a nucleic acids decontamination solution (NucleoClean^TM^ Decontamination Solution, Merck Millipore n° 3097S) prior to mix preparation. A general PCR protocol with slight modifications was applied on all vertebrate gDNA (see Supplementary dataset S22). PCR products quality and length were assessed by gel electrophoresis and negative and positive controls were always included. Whenever possible, more than one sample per species has been tested (as listed in Supplementary dataset S1).

### Amplification of pHTT

The *pHTT* belonging to *Callithrix jacchus* was amplified according to the genome information (Ensembl release 91, C_jacchus3.2.1). The reaction was performed in a total volume of 15 μl with 1 μl of genomic DNA (buffer HF 1×, MgSO_4_ 0.8 mM, dNTPs 0.2 mM, Primer FW 0.35 μM, Primer RV 0.35 μM, Taq Platinum HF 5 U/μl, DNA ~100 ng/μl; Primer FW: 5′-GCGACCCTGGAAAAGCTGAT-3′; Primer RW: 5′-TGGTCAGGGCTTGCAGAAG-3′). The amplification consisted of 10′ at 96 °C; 36 cycles of 45″ at 96 °C, 30″ at 58 °C, 1′30″ at 68 °C; 10′ at 68 °C. PCR products quality and length (~1100 bp) were assessed by gel electrophoresis. PCR products were sequenced, using Sanger technology, either directly or after cloning.

### Cloning and sequencing of PCR products

TOPO^®^ TA Cloning^®^ Kit (Life Technologies, CAT n° K4575) was used to clone the PCR amplification product for direct sequencing. A commercial kit (Qiagen, CAT n° 27106) was used to perform the DNA mini-preparation. PCR product purification and sequencing reactions were carried out on ABI machines according to manufacturer specification either by us or MWG Operon (Eurofins Genomics, Ebersberg, Germany) and GATC Biotech AG (Costance, Germany). PCR products were Sanger sequenced either directly or after cloning. Technical replicates of sequencing were performed. More than one sample per species, when available, has been sequenced (as listed in Supplementary dataset S1).

### Alignment of exon1 orthologs

Multiple sequence alignments (MSA) were conducted with Clustal Omega [29]. Alignment refinements were performed manually in SeaView 4 (http://doua.prabi.fr/software/seaview), CLC Sequence Viewer (www.qiagenbioinformatics.com) and Geneious version 9 (www.geneious.com) (the overall alignment used is available as Supplementary dataset S2, as well as taxa subgroups alignments are available as supplementary nexus files).

### Test for purifying selection

The tests for negative/positive selection were performed on MSA organized as Nexus files with custom evolutionary trees. For all analyses, MSAs including gap (e. g. CCN region in mammals) and unknown regions (i.e., primer bind and LQ base calling) were employed. Phylogenetic tree topologies for sampled organisms were obtained from the TimeTree.org resource (http://www.timetree.org), exported in the Newick format and embedded in the Nexus files containing the MSAs. To estimate the extent of pervasive purifying selection at individual sites we used three different methods: the Single-Likelihood Ancestor Counting (SLAC) method [30], the Fixed Effects Likelihood (FEL) method [30], and the Fast Unconstrained Bayesian AppRoximation (FUBAR) method [31]. SLAC is the simplest inference legacy method: it is based on counting how many synonymous and non-synonymous changes have occurred at each site over evolutionary time and uses the binomial distribution as a statistical test [30]. FEL instead estimates a value for dN and dS at each considered site testing it using a maximum likelihood approach and a chi squared distribution [30]. FUBAR is particularly efficient on larger datasets (high number of sequences) and takes a Bayesian approach to selection inference developed in the context of document classification (latent Dirichlet allocation) [31]. Finally, FUBAR also calculates an a priori probability over a specified grid of d*N* and d*S* values thus defining a space of probability over a wide range of selection scenarios.

Calculations were performed using implementations on the Datamonkey Servers (version 2, www.datamonkey.org/) and/or a local instance of the HyPhy software (version 2.2.4), and all methods were used with built-in defaults parameters. The taxa list of the species initially considered and the Nexus input files used for the purifying selection analyses after pruning (HyPhy discards identical sequences) are available as supplementary data files.

### Retrieval and statistical testing of phylogenetically conserved polyCAG/CAA genes

All human (genome assembly hg19) coding sequence (CDS) transcripts with at least four consecutive glutamines were downloaded from Ensembl and for each of these genes the longest CDS was considered. Sequence orthologs were retrieved from Ensembl for all available mammalian genomes and aligned at the protein level using Clustal Omega [29]. Codon alignments were reconstructed using custom Python scripts. For each non-human sequence, the region of the alignment corresponding to the human polyQ tract was examined and the longest pure glutamine stretch overlapping the human stretch or conjoined alignment gaps was considered. Subsequently, the longest Q stretch (LQ) and the longest non-interrupted CAG interval (LNI) was calculated for each of these stretches. To reduce the impact of phylogenetic structure, only taxa that had a curated *HTT* sequence were considered. Conserved tracts were selected based on their presence in more than five primate and five non-primate genomes for which HTT sequences were available (considering those species for which the HTT sequence was also available). Only polyQ tracts that displayed at least two different polyCAG/CAA lengths were tested.

A standard score (*z*-score)-like statistic was calculated for LQ, LNI, and PQ (purity of the polyQ tract, i.e., CAG/CAA proportion) for each polyQ tract [e.g., (LQ—mean LQ for orthologous tracts)/standard deviation of LQ for orthologous tracts], and the frequency distributions of these scores were considered.

### HTT and pHTT comparative analysis

General Time Reversible (GTR) model (with four discrete gamma distributed substitution rate categories) was used to estimate numbers of substitutions in *Callithrix jacchus HTT* and *pHTT* sequences under maximum likelihood criterion (using PAUP [32]) with *Aotus nancymaae HTT* sequence as outgroup. All rate parameters were estimated on the alignment and tree topology. The rates of substitutions are represented as the ratio between observed and expected substitutions, according to the length of each exon. Expected values were calculated on the basis of the total number of substitutions and the total length of the alignment, and scaled to the exon lengths.

### Mouse embryonic stem (mES) cell lines

We used mouse E14 ES cell lines [33]: parental (+/+), heterozygous (+/−), or homozygous (−/−; RMCE/−) knock-out for the *HTT* gene. RMCE/− cell line was used to generate HTT knock-in cell lines (0Q, 2Q, 4Q, 7Q, 10Q, 13Q, and 17Q). Q banding karyotype analysis performed by Integrated Systems Engineering srl (ISENET) on ±/+, −/−, and RMCE/− cell lines confirmed normal karyotype. The expression of *OCT4* and *SOX2* pluripotency markers was confirmed by immunofluorescence in all the cell lines generated. We also used R1 mES cell lines [34] expressing the wild-type *HTT* gene (*Hdh*^*+/+*^) or being homozygous knock-out for the gene (*Hdh*^*ex4–5*^), in which both alleles of the *HTT* gene were inactivated by deletion of exons 4 and 5 [35]. All transgenic cells were derived by the transfection of pCAG constructs in *Hdh*^*ex4–5*^ cells. Both *Hdh*^+/+^ and *Hdh*^*ex4–5*^ were analysed for karyotypic abnormalities (Q-banding) and they showed several common [36] chromosomal alterations at the cell population level (the most frequent were duplications of chromosomes 1, 8, 11, and 14).

### Mouse ES cell culture

ES cells were maintained in Glasgow minimal essential medium supplemented with 10% heat-inactivated fetal bovine serum (vol/vol, EuroClone, REF ECS0186L), 0.1 mM β-mercaptoethanol (Gibco, REF #31350-010), 100 μM non-essential amino acids (Gibco, REF #11140-035), 1 mM sodium pyruvate (Gibco, REF #11360-039), 2 mM l-glutamine, 100 U/ml penicillin, 100 μg/ml streptomycin (EuroClone, REF ECB3001D) and 1000 U/ml murine leukemia inhibitor factor (LIF, ESGRO) (Millipore, REF ESG1107) in gelatinized tissue culture flasks. Cells were passaged every 2 days after dissociation with 0.05% trypsin-EDTA (vol/vol) (Gibco, REF 15400-054).

### *HTT* knock-out

gRNA2p and gRNA5i (supplementary dataset S23), respectively annealing 250 bp upstream and 430 bp downstream *HTT* exon1, were used to drive its precise deletion in E14 +/+ mES cells [37]. Alt-R® CRISPR-Cas9 tracrRNA (IDT) and the two designed Alt-R® CRISPR-Cas9 crRNAs were separately assembled in vitro and added to the Alt-R® *S.p*. Cas9 nuclease (IDT) following manufacturer’s instruction. A mix containing 1.8 pmol of each ribonucleoprotein (RNP) complex was introduced by reverse lipofection in 320,000 E14 +/+ mES cells using Lipofectamine® RNAiMAX (Thermo Fisher). Clones were isolated and screened by PCR for the presence of the small expected knock-out band with oligos crHTTexon1kofw and crHTTexon1korev (supplementary dataset S23). Three primers-based PCR assay enabled to better discriminate between homozygous and heterozygous clones (Fig. S7A, B and Supplementary dataset S23). Two validated clones for each genotype were exposed to neural rosette assay.

### *HTT* knock-in

Two gRNAs cutting 1.5 kb upstream (Cas9 HAL) and 2 kb downstream (Cas9 HAR) *HTT* exon1 were used to integrate the RMCE cassette by homologous-directed recombination (HDR) (supplementary dataset S23). Two million E14 +/+ mES cells were nucleofected using Amaxa^TM^ mES nucleofector kit (Lonza) with a mix containing 20 pmol of each in vitro assembled RNP complex, 10 μl nucleofector enhancer (IDT) and 1 μg RMCE-Htt-PuroTK donor cassette (synthetized by GenScript and cloned in pUC57). The HDR donor vector contained 1 kb homology arms and the RMCE cassette, that comprises the FRT and F3 sites specific for the Flp recombinase and the positive/negative selectable marker PuroRΔTK under the human PGK promoter (supplementary dataset S23). Cells were seeded 35,000 cells/cm^2^ and grown in presence of 1 μM SCR7 ligase IV inhibitor for 24 h after nucleofection to promote repair by HDR. Clones resistant to 2 μg/ml puromycin were isolated and screened by PCR for the integration of the RMCE cassette without random insertion of the donor vector at first and subsequently for genotyping the second allele (primer pairs used listed in supplementary dataset S23).

We selected one RMCE/− clone in which we integrated by Flp-mediated recombination 0Q, 2Q, 4Q, 7Q, 10Q, 13Q, and 17Q RMCE constructs (synthetized by GenScript and cloned in pUC57 vector) to reconstitute the whole genomic portion removed by the PuroRΔTK RMCE cassette. To this aim, 2 million RMCE/− cells were nucleofected using Amaxa^TM^ mES nucleofector kit (Lonza) with 4 μg RMCE donor construct (either 0Q, 2Q, 4Q, 7Q, 10Q, 13Q, or 17Q) and 4 μg pCAG-Flpe:GFP (Addgene, plasmid #13788). Cells were seeded at low density (4–8000 cells/cm^2^) and exposed to 1 μM ganciclovir selection 72 h after nucleofection. Recombination efficiency resulted to be higher than 60% for all constructs. Clones were individually validated and two clones for each genotype were exposed to neural rosette assay.

### Clone validation

*Sequencing of HTT locus*. PCR products from *HTT locus* were purified using QIAquick PCR-purification Kit (QIAGEN) and Sanger sequenced (GATC—Eurofins).

*HTT expression*. RNA was isolated from clones of interest with TRIzol reagent according to the manufacture’s instruction (Life Technologies) and reverse transcribed with iScript cDNA Synthesis Kit (Bio-Rad). The presence of HTT mRNA was evaluated using primers amplifying exon 3–6 cDNA (supplementary dataset S23) and HTT expression was checked by western blot analysis using D7F7 antibodies (Cell Signaling Technology).

### Transgenic mouse ES cell lines

*Constructs*. The N-terminal portions of mouse (*Mm*) HTT were designed according to previously published HTT MSA [17] and cloned in pCAG constructs: pCAG Mm 0Q, pCAG *Mm* 2Q, pCAG *Mm* 4Q, pCAG *Mm* 7Q, pCAG *Mm* Q3PQ3, pCAG *Mm* Q3Q(CAA)Q3.

*DNA transfections. Hdh*^*ex4-5*^ ES cells were plated at a density of 2 × 10^4^ cells per cm^2^. After 24 h, cells were transfected with different expression vectors (10 μg) and Lipofectamine 2000 (Thermo Fisher Scientific, REF 11668027). Cells were selected with puromycin (2 μg ml^−1^) 24 h after lipofection for 10 days. Western blot and immunofluorescence were performed to determine the expression of the transgene.

### Protein lysates and western blots

Cells were lysed in RIPA buffer (50 mM Tris-HCl pH 8, 150 mM NaCl, 0.1% SDS, 1% nonidet P40, 0.5% sodium deoxycholate, wt/vol) with 1 mM PMSF and protease inhibitor (Thermo Scientific, REF 1861281). Lysates were cleared by centrifugation at 12,000×*g* and 4 °C for 30 min. The resulting supernatant was collected. Protein concentration was determined with the Pierce-BCA Protein assay kit (Thermo Scientific REF 23225) and 30 μg were loaded on a 7.5% SDS-PAGE gel. Separated proteins were transferred to a nitrocellulose membrane, blocked with 5% non-fat dry milk (wt/vol, Biorad, REF 170-6404) in Tris-buffered saline (TBS) and 0.1% Tween-20 (vol/vol), and incubated with primary antibody at room temperature (20–23 °C) for 3 h. After washing, filters were incubated for 1 h at room temperature with a secondary antibody (peroxidase conjugate, Biorad, 1:3000) and then washed three times with TBS and 0.1% Tween-20. The Clarity Western ECL Substrate (Biorad, REF 170-5061) was used to visualize immunoreactive bands by chemiluminescence detection with ChemiDoc MP Imaging System (Biorad).

### Mouse ES cell monolayer differentiation

ES cells were dissociated and plated onto 0.1% gelatin-coated tissue culture dishes at a density of 1.6–2.4 × 10^4^ cells per cm^2^ in N2B27 medium. N2B27 medium was a 1:1 mixture of DMEM/F12 and Neurobasal medium containing 1:200 N2 supplement (Gibco, REF 17502-048), 1:100 B27 supplement (Gibco, REF 17504-044) and 0.1 mM β-mercaptoethanol (Gibco, REF 31350-010). Medium was renewed every 2 days. Neural rosettes formed by progenitor cells were analysed after 8–9 days of differentiation.

For the terminal differentiation process, on day 12–14 neural progenitors were dissociated with Accutase (Millipore, REF SF006) and plated onto 10 ng/μl Poly-d-Lysin (Sigma Aldrich, REF P6407) and 3 ng/μl Lamin (Thermo Fisher, REF 23017015) coated tissue culture dishes at density of 1 × 10^5^ cells per cm^2^ in N2B27 post re-plating medium. N2B27 post re-plating medium was a 1:4 mixture of DMEM/F12 and Neurobasal medium containing 1:100 N2 supplement (Gibco, REF 17502-048), 1:50 B27 supplement (Gibco, REF 17504-044), and 30 ng/ml Brain-derived neurotrophic factor (PeproTech, REF 450-02). Medium was renewed every 2 days. Terminal differentiated cell cultures were analysed after 28 days of differentiation.

### Immunofluorescence

Cells were fixed in 4% paraformaldehyde for 15 min at 20–23 °C. Cells were permeabilized and blocked in blocking buffer containing PBS with 0.5% Triton X-100 (vol/vol) and with 5% fetal bovine serum (vol/vol) for 1 h. Primary antibodies were diluted in blocking buffer and applied overnight at 4 °C. After three washes in PBS, appropriate secondary antibodies, conjugated to Alexa fluorophores 488 or 568 (Molecular Probes, Invitrogen) and diluted 1:500 in blocking solution, were applied for 1 h at room temperature (20–23 °C). Cells were incubated for 10 min with Hoechst 33258 (5 μg/ml, Molecular Probes, Invitrogen). Images were acquired with a Leica DMI 6000B microscope (equipped with LAS-AF imaging software) or with an IN Cell Analyzer 6000 (GE Healthcare Life Sciences) and processed with the software ImageJ (US National Institutes of Health) and CellProfiler (vers.2.1.1).

### Rosette and neuron quantification

*Rosette quantification*. In the E14 cell differentiation experiments, the rosette lumen areas were quantified on day 8–9 of neural differentiation after staining for NESTIN and PALS1. CellProfiler software was used to automatically quantify rosette lumens (PALS1 positive areas). In the R1 cell differentiation experiments, rosettes were quantified on day 7 of neural differentiation after staining for NESTIN and ZO1. For the measurement of the % of NESTIN+ cells inside rosettes a previously established protocol was applied [18]. For rosette and lumen size, the contour of each rosette (as highlighted by marker NESTIN) and lumen (as highlighted by marker ZO1) was outlined using ImageJ software and two lists of regions of interest (ROIs) were measured to calculate rosette mean areas and lumen mean areas, respectively.

*Neuron quantification*. In the E14 cell terminal differentiation experiments, the neurons were analysed on day28 of neural differentiation after staining for MAP2, TUBB3, P27, and NEUN. CellProfiler software was used to automatically quantify MAP2 and TUBB3 positive areas as well as P27 and NEUN positive cells. The relative number of P27 and NEUN positive cells was calculated as a percentage of total Hoechst positive cells.

### Antibodies

The antibodies and dilutions used for immunofluorescence and western blotting were: monoclonal antibody to Huntingtin (Millipore, Mab2166; immunofluorescence, 1:100; western blotting, 1:1000; Cell Signaling Technology, D7F7, western blotting, 1:1000), monoclonal antibody to NESTIN (Millipore, Mab353; immunofluorescence, 1:200), polyclonal antibody to ZO1 (Life Technologies, REF 402300; immunofluorescence, 1:200), polyclonal antibody to PALS1 (Proteintech, 17710-1-AP; immunofluorescence, 1:500), monoclonal antibody to OCT4 (Santa Cruz, sc-5279; immunofluorescence, 1:100), polyclonal antibody to SOX2 (Millipore, AB5603; immunofluorescence, 1:200), polyclonal antibody to TUBB3 (BioLegend, 802001, immunofluorescence, 1:1000), monoclonal antibody to MAP2 (BD, 556320, immunofluorescence, 1:500), monoclonal antibody to P27 (Cell Signaling Technology, 3698, immunofluorescence, 1:1000), monoclonal antibody to NEUN (Millipore, Mab377; immunofluorescence, 1:100).

### RNA extraction (for real-time qPCR or RNA-seq analyses)

RNA was isolated using TRIzol reagent according to the manufacture’s instruction (Life Technologies). RNA was quantified with Nanodrop and then the integrity was evaluated. Potential contaminating DNA was removed by DNA-free kit (Ambion). RNA was reverse transcribed using iScript cDNA Synthesis Kit (Bio-Rad).

### Real-time qPCR

qPCR was performed using the CFX96 Real-Time System (Bio-Rad). All reactions were performed in 15 μl containing 50 ng (or 100 ng for SHH and PLAGL1) cDNA and SsoFast EVAGreen Supermix (Bio-Rad). Primer pairs used were listed in supplementary dataset S23.

### Apoptosis assay

Cells were seeded in triplicate in two 96-well plates, at 16 × 10^3^ cells per well. After 24 h incubation at 37 °C, cells received 100 μl of freshly prepared GMEM + LIF without serum. At 48 h after plating, 100 μl of CellTiter Reagent (Promega, REF G7570) was added to all wells. After 10 min of incubation at 20–23 °C, luminescence was measured with a Veritas-MicroplateLuminometer (Turner Biosystems) to detect cell viability. Caspase-Glo 3/7 Reagent (100 μl, Promega, REF G8091) was added to all parallel wells. After 30 min of incubation at 20–23 °C, luminescence was recorded with a Veritas-MicroplateLuminometer (Turner Biosystems) to detect CASPASE-3/7 activity. Each experiment was independently repeated at least three times. Results are presented as the ratio of CASPASE-3/7 luminescence/cell viability luminescence recorded for each cell line.

### Statistical analyses of biological assays

Data from the quantification of the rosette, neuron and apoptosis assays were analysed with one-way ANOVA by using GraphPad Prism (version 7). Posthoc comparisons between cell lines were performed by applying Tukey or Bonferroni procedure. Correlation analysis between *Q* lengths and phenotypes were calculated by linear regression analysis (Pearson’s and Spearman’s tests).

### RNA sequencing and analyses

*Sequencing*. RNA-seq was performed on 0Q, 2Q, 4Q, and 7Q cell lines (three biological replicates per cell line) at the rosette (day 8) and neuronal (day 29) stage. Stranded polyA libraries were sequenced on the Illumina HiSeq 4000 platform, with an average depth of 40 million 150 bp paired-end reads per sample.

*RNA-seq quality control and mapping*. The quality of the reads was tested with MultiQC [38] before and after a trimming step performed using Trimmomatic [39]. Low-quality reads or reads shorter that 50 bases were removed. Adapters and low-quality bases (quality < 3) in both leading and trailing ends of the reads as well as any four consecutive bases with an average quality per base less than 15 were also removed. Reads were then aligned to the mouse reference genome (GRCm38.98, with gene annotation GENCODE M25) using STAR v2.5.2b [40] with default parameters.

*RNA-seq data processing*. Differential expression analysis was performed with DESeq2 [41]. To take into account experimental batches, replicates were added as additional variables and included into the design formula.

At the rosette and neuronal stages all the possible pairwise comparisons were performed (0Q vs. 2Q, 0Q vs. 4Q, 0Q vs. 7Q, 2Q vs. 4Q, 2Q vs. 7Q, and 4Q vs. 7Q). Genes were considered differentially expressed if the adjusted *p*-value (Benjamini Hochberg) was lower than 0.05 and the absolute log2 fold change was higher than 1. Genes with low base mean [<1 CPM (counts per million)] were not considered in the analysis.

*Functional analysis*. Enrichment analysis for Gene Ontology (GO) terms was performed using TopGO [42], focusing on the “Biological Process” category. GO terms with Fisher test corrected *p*-values <0.05 were considered as significant. The GOplot package [43] was used to generate circular plot and compute *z*-scores (number of upregulated genes minus the number of downregulated genes divided by the square root of the count).

*“Jaccard similarity” index*. The analysis was performed by comparing the top 100 Differentially Expressed Genes (DEGs) between rosettes (early stage) and neurons (terminal stage) of 0Q, 2Q, 4Q, and 7Q neuronal cultures with the top 100 unique DEGs from bulk RNA-seq data of human fetal cortical (CTX), striatal (Lateral Ganglionic Eminence, LGE) and pallidal (Medial Ganglionic Eminence, MGE) tissues [44].

## Results

### The HTT polyQ tract is under purifying natural selection

**Fig. 1:**
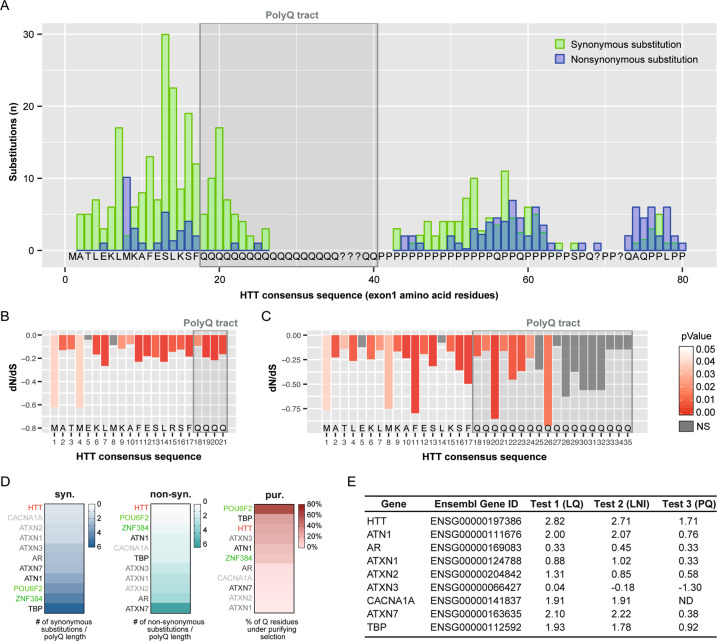
Effects of natural selection on *HTT* exon1. **A** Synonymous and non-synonymous substitutions counted with the codon-based maximum likelihood method SLAC on the multiple alignment of 163 unique, non-redundant sequences from vertebrates (*n* = 158), and basal species (*n* = 5). A time-tree was used as backbone for calculations. Synonymous (green) and non-synonymous (blue) substitution counts are shown for each codon (consensus sequence in the plot); the gray shaded box highlights the polyQ tract. **B** d*N*/d*S* ratios determined by FUBAR method for the multiple sequence alignment (MSA) subset of bony fishes, turtles, crocodiles, and birds (*n* = 84 species) where four glutamine-encoding codons can be unambiguously aligned. Consensus sequence of *HTT* exon1 is shown for reference; the gray shaded box highlights the polyQ (4Q) tract. **C** d*N*/d*S* ratios determined by FUBAR method for the MSA of mammals (*Q* ≥ 4, *n* = 74 species), where the number of *Q* encoding codons is variable. HTT N-terminal consensus sequence is shown for reference. The gray shaded box highlights the polyQ (*Q* ≥ 4) tract. The orientation of peaks in plots **B** and **C** indicates the direction of selection (downward = purifying/negative; upward = diversifying/positive); peaks height indicates the strength of selection (d*N*/d*S* values) and peak’s color (shades of red) shows the statistical significance level. **D** Heatmaps showing comparison of the polyQ stretch conservations for nine polyQ disease-associated genes (*HTT* in red, others in gray scale) and two genes not associated with any type of diseases (*POU6F2* and *ZNF384*, green) across taxa. The heatmaps display the value of synonymous substitutions over Q length ratio (syn.), the value of non-synonymous substitutions over Q length ratio (non-syn.) and the fraction of Q residues under significative purifying selection (pur.). **E** Table showing the comparative analysis of the disease-associated polyQ proteins. *Z*-score values for the longest Q stretch (LQ), for the longest non-interrupted CAG interval (LNI) and for the CAG/CAA proportion (PQ) of the nine human disease-associated genes, extracted from the results of the three analyses (test 1, 2, and 3) described in Fig. S6B–D.

We started by investigating whether long-term patterns of conservation and change for HTT CAG repeats have been subjected to selective pressures. To this aim, we assembled a Multiple Sequence Alignment (MSA) of HTT exon1 sequences from more than 200 chordate species—108 de novo sequenced orthologs and 101 sequences retrieved from online databases or published studies (Supplementary datasets S1 and S2). Our MSA displays variation in the length of the HTT polyQ tract across chordate groups (Fig. S1). To understand whether selective pressures contributed to these patterns, we measured first the ratio of nonsynonymous to synonymous substitutions (d*N*/d*S* ratio) for each position of this *locus*. A low rate of between-species nonsynonymous substitution—that is a nucleotide mutation that alters the amino acid sequence of a protein—reflects the action of purifying selection within species to eliminate mildly deleterious mutations and preserve the tract intact. In our analyses we estimated the substitution occurrences for 163 non-redundant (unique) highly confident exon1 sequences (Supplementary dataset S3). Importantly, we found that only two of the 291 inferred non-synonymous substitutions within the exon1 *locus* fall within the polyQ tract (Fig. 1A).

Second, for a more rigorous conservation/selective pressure analysis on the polyQ tract, we quantified the d*N*/d*S* ratio. When there are substantial variations in CAG repeat length between species—driven by repeat expansion/contraction events—the region could not be reliably aligned because the *N*th CAG (or Q) in one species could not be orthologous to the *N*th CAG repeat in a different species. Being aware of this possible limitation, we assessed d*N*/d*S* ratio on non-mammalian sequences with stable 4Q repeats (*n* = 84 species) (Supplementary dataset S4)—as the absence of indels allows confident alignment across their entire length. The most commonly used approaches to assess positive or negative (purifying) selection through the d*N*/d*S* ratio across the phylogeny are SLAC, FEL [30], and FUBAR [31], which are based on different algorithms and statistical tests (see “Methods” section). All these approaches (FUBAR, Fig. 1B; SLAC and FEL, Fig. S2A, C) showed that purifying selection acts on the four Q-encoding codons. In particular, the downward columns in Fig. 1B indicate that the four Qs, similarly to the first 17 aa, are under purifying selection. Despite the possible limitations of aligning sequences bearing different CAG lengths, the three different approaches (FUBAR, SLAC, and FEL) suggest consistent purifying selection acting also on the polyQ tract of the mammalian sequences with *Q* ≥ 4 (*n* = 74 species) (Fig. 1C, S2B, D and Supplementary dataset S5). Furthermore, possible evolutionary scenarios (negative, neutral, and positive selection) were evaluated by FUBAR method on the *HTT* exon1 as a whole (Fig. S3). The distribution of substitution rates probability in Fig. S3A, B displayed that the higher (red) probability for *HTT* exon1 fell exclusively into the negative selection area (Fig. S3A, B) indicating that *HTT* exon1, as a whole, is under purifying selection.

This is the first time a wide set of orthologs has been taken into consideration to represent aspects of HTT’s unique evolutionary history, among the polyQ-containing proteins under selective pressures [45], and to demonstrate that *HTT* exon 1 as a whole and its polyQ tract underwent purifying selection.

### The HTT polyQ tract has been subjected to the strongest selective pressures throughout evolution compared to other polyQ-containing genes

In order to investigate whether similar selective pressures might also act on the other polyQ, we tested the polyQ stretch of Ataxin-3 (ATXN3) which is associated with the second most common polyQ disease, Spinocerebellar ataxia type 3 (SCA3) [46]. To this end, we generated an alignment of the ATXN3 polyQ *locus* from 53 taxa for which both ATXN3 and HTT sequences were available, thus sharing phylogenetic relationships and timescales (Supplementary datasets S6–8). Figure S4A, B show, for the same taxa, the distribution of substitutions around the polyQ region of ATXN3 and HTT, respectively. While 53 synonymous and 43 non-synonymous substitutions were found in the ATXN3 polyQ stretch, also in this sample only 21 synonymous substitutions were observed in the HTT polyQ stretch. FUBAR identified that 12.5%, for ATXN3, and 44.4%, for HTT, of Qs were under significant purifying selection (Fig S4C, D). This suggests that the HTT polyQ tract is under stricter selective pressures compared to the ATXN3 polyQ *locus*.

We then performed selective pressure analysis on the polyQ tract of the other seven brain disease-related proteins [47], and of two proteins not-associated with any disease (POU6F2 and ZNF384) (Fig. 1D and S5 and Supplementary datasets S9–17). Comparative SLAC analysis revealed that the HTT polyQ tract is the first in terms of low presence of synonymous and non-synonymous substitutions (Fig. 1D, and S4A, B and S5A). The analysis also revealed that this tract has one of the highest percentages of Q residues under significant purifying selection, ranking third after POU6F2 and TBP (Fig. 1D and S4C, D and S5B).

Recent findings showed that HD pathogenesis is influenced by the size of the uninterrupted CAG repeat sequence [6]. In particular, it is known that the stretch of CAG in the human *HTT* gene is interrupted by a penultimate CAA codon. Studies from the last few years have demonstrated that the loss of this interruption, or the duplication of the ultimate CAA-CAG codons, are associated with an earlier or later—respectively—age of disease onset [6, 7], suggesting CAG purity is a causal driver of CAG somatic instability in HD [6, 7]. We therefore decided to explore mammalian genomes to compare *HTT* CAG purity and length with those of other genes encoding polyQ-containing proteins. We retrieved from the Ensembl database 248 sets of mammalian protein orthologs, containing at least one tract of four or more consecutive Qs for which length variability among mammalian species is observed, and collectively obtained 367 polyQ tracts because some proteins bear multiple polyQ tracts (Fig. S6A). For each individual polyQ tract, we calculated the *z*-scores (see methods) for (*i*) length of polyQ (LQ), (*ii*) longest uninterrupted CAG repeat (LNI), and (*iii*) polyQ tract “purity” (proportion of CAG/CAA codons) with respect to orthologous sequences. We found that the human HTT polyQ tract behaved as an outlier both in terms of LQ (Fig. S6B and Supplementary dataset S18) and LNI (Fig. S6C and Supplementary dataset S18), as shown by its position in the right-most end tail of the distributions. The human HTT polyQ tract also displays an exceptionally low presence of CAA “impurity” compared to polyQ stretches of other proteins (Fig. S6D and Supplementary dataset S18). Indeed, human *HTT* displays the highest length and purity of the CAG tract compared to orthologs of known polyCAG/CAA disease-associated genes (Fig. 1E), such as ATXN3 (as also expected from the previous polyQ conservation analysis; Fig. S4A, C).

These results indicate that strong purifying selection acts on the CAG/CAA repeat region of the *HTT* gene to maintain its capacity to encode glutamine residues. Our data also indicates that the human HTT polyQ encoding tract stands out for its length and purity of CAG codons with respect to other polyQ tracts in mammals. In conclusion, this level of conservation and purity in the HTT polyQ could have paved the way for its evolutionary expansion [7, 48–50].

### The structure of a de novo identified HTT pseudogene confirms that purifying selection acts on the CAG repeat region

**Fig. 2:**
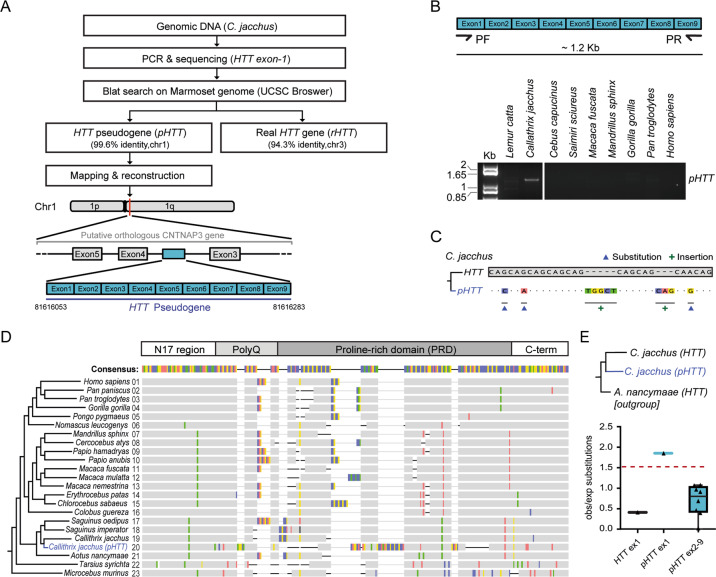
*HTT* gene and pseudogene comparison in *Callithrix jacchus*. **A** Schematic pipeline of *pHTT* identification. **B** PCR analysis of *pHTT* in primates. See Supplementary dataset S24 for raw gel image. **C** Variations observed in the CAG/CAA tract of *pHTT* analysed as compared to the CAG/CAA tract of the *Callithrix jacchus HTT* gene: G → C, G → A, A → G substitutions (▲); TGGCT and CAG insertions (+). **D** Graphical representation of the differences (substitutions and indels; highlighted with colors) between the *HTT* orthologous exons1 in primates (1–19 and 21–23; corresponding to black branches in the tree) and the *pHTT* of *Callithrix jacchus* (20; blue branch) as compared to the consensus sequence. **E** Comparison of *HTT* exon1 and *pHTT* exons1–9 instability of *Callithrix jacchus* measured as the ratio of number of substitutions observed and expected (relative to number on amino acids per exon) with respect to the outgroup *HTT* sequence (*Aotus nancymaae*). Dots (triangles) represent the ratio of observed on expected (obs/exp) substitutions *per* exon.

In our sequencing effort, we also serendipitously identified a processed *HTT* pseudogene (*pHTT*) in the marmoset *Callithrix jacchus*, which had escaped previous identification [51], and whose sequence allows the description of the mutational dynamics of this DNA sequence in the absence of selective constraints. *Callithrix jacchus pHTT* consists of the first nine *HTT* exons and lays within the third intron of the *CNTNAP3* gene (Fig. 2A, B and Supplementary dataset S19). Three point-mutations (one non-synonymous) and several indels, including a frameshift inducing insertion, were observed between the CAG tract of *Callithrix jacchu*s *pHTT* and *HTT* (Fig. 2C). Numerous indels were also observed between the proline-rich domain (PRD) of the *Callithrix jacchus pHTT* and other primate *HTT* sequences (Fig. 2D). These observations likely reflect the absence of purifying selection acting on the pseudogene sequence and hint at the underlying mutability of sequences encoding the PRD. Interestingly, *pHTT* exon1 in *Callithrix jacchus* is far more prone to point substitutions than the rest of the pseudogene exons, consistent with studies indicating that point substitution rates are higher around indel sites [52] (Fig. 2E and Supplementary dataset S20) and further attesting to the purifying selection acting on *HTT* exon1.

### The length and purity of the polyQ tract affects HTT neuronal function

**Fig. 3:**
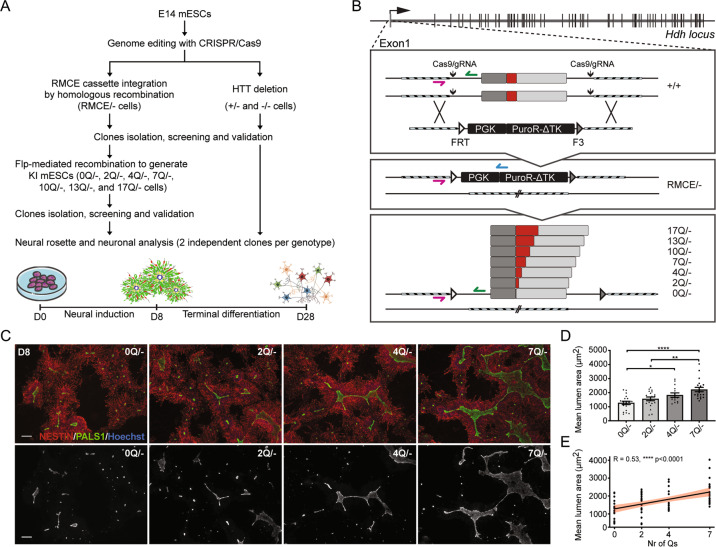
PolyQ tract impacts the formation of neural structures. **A** Pipeline for the generation and characterization of HTT knock-in and knock-out E14 mESCs. **B** Genome editing strategy used to produce knock-in mESCs. CRISPR/Cas9 with two gRNAs, was used to insert the RMCE cassette replacing the exon1 on one allele and to delete HTT exon1 on the other allele, thus generating the RMCE/− cell line. The RMCE cassette contains the positive/negative selectable marker PuroR-ΔTK, under PGK promoter, flanked by FRT and F3 recombination sites, which were used to direct the integration of modified exons 1 with 0, 2, 4, 7, 10, 13, and 17 Q repeats by Flp recombinase. gRNAs and oligos sequences are reported in Supplementary dataset S23. **C** Representative images of rosette/lumen phenotype in 0Q/−, 2Q/−, 4Q/−, and 7Q/− cells stained for PALS1 and NESTIN at day 8 of neural induction. **D** Mean lumen area (μm^2^) of 0Q/−, 2Q/−, 4Q/−, and 7Q/− cell cultures. *P < 0.05, **P < 0.01, ****P < 0.0001, one-way ANOVA test followed by Tukey. **E** Linear regression analysis and confidence interval between Q length and lumen size in 0Q/−, 2Q/−, 4Q/−, and 7Q/− cell cultures. Pearson correlation coefficients (R) and *P*-values are reported on the plots. Data in **D** and **E** are expressed as mean ± SEM from *n* ≥ 4 independent experiments. Each dot represents the mean lumen area per well testing a pool of two clones for each edited cell line (see Supplementary dataset S25 for raw data). The scale bars correspond to 50 μm.

Since long polyQ tracts are enriched in genes with neuronal functions [53], and since HTT is implicated in neurogenesis [18, 54], we looked for evidence of polyQ activity itself in in vitro neural cell assays. We therefore genome-edited E14 mES cell line to knock-in 0, 2, 4, 7, 10, 13, or 17 Qs in the endogenous *HTT* gene (Fig. 3A, B). 7Qs are found in the mouse HTT protein ([55, 56] and Fig. S1). First, we integrated a Recombinase-Mediated Cassette Exchange (RMCE) cassette at the exon1 *locus* by CRISPR-driven homologous recombination, in parallel with the deletion of the other *HTT* allele to create RMCE/− cells. Flp-mediated exchange of the RMCE cassette was then used to integrate modified exons (Fig. 3A, B). Heterozygous (+/−) and homozygous (−/−) HTT knock-out control cell lines were also generated (Fig. S7A–F). Editing was checked by sequencing and PCR (Fig. S7G, H). HTT expression was evaluated by RT-PCR and western blot (Fig. S7I, J) and pluripotency maintenance by immunofluorescence and qPCR for OCT4, NANOG, and SOX2 (Fig. S7K–N).

To evaluate whether a neural potential is associated with the polyQ tract, we initially exposed the cell lines to neural induction and measured their capacity to form organized radial structures with a central lumen, known as neural rosettes, that express many of the proteins found in the developing neuroepithelium in vivo [18, 57] (Fig. 3A). As expected, −/− and RMCE/− mES cells formed NESTIN + rosettes with significantly reduced lumen size, as revealed by immunostaining against the cell polarity protein PALS1, compared to +/+ and +/− cells. This finding confirmed previous results indicating that the loss of normal HTT disturbs rosettes formation (Fig. S8A, B) [18]. Importantly, we observed that neural progenitors expressing the entire HTT protein lacking only the single polyQ tract (0Q cells) form rosettes with a significantly reduced lumen size with respect to 7Q cells that carry the wild-type length of the murine tract (Fig. 3C, D), indicating that the HTT polyQ tract itself is biologically active in a rosette assay. Neural progenitors with 10, 13, and 17 Qs formed rosettes of the same lumen size as 7Q culture (Fig. S9A, B), suggesting that, in our mouse cell assay, rosette formation reaches a plateau when at least seven Qs—which is the size in mice—are present in the murine HTT context. As a consequence, we restricted our analyses of a possible correlation between the polyQ length and neural potential focusing on biological, imaging and transcriptional analyses of mES cell lines bearing 0, 2, 4, and 7 Qs. We found that a stepwise increase of Qs from 0 to 7 increased the rosette lumen size. In fact, 0Q/− cells displayed lumens that were significantly smaller than those in 4Q and 7Q cells, while 2Q lumens were significantly smaller than those in 7Q lines (Fig. 3C, D). In this set of cells, a linear regression analysis showed a significant correlation between the number of Qs and the mean lumen area of the rosettes, especially from 0 to 7Qs (Fig. 3E), suggesting that tracts with an increasing number of Qs are progressively more active in shaping the spatial organization of neural progenitors.

Similar results, regarding rosette formation, were obtained with transgenic R1 *Hdh*^*ex4-5*^ mES cells lacking HTT [35] and overexpressing a large fragment of mouse HTT corresponding to the first 548aa portion carrying 0, 2, 4, or 7 Qs (Fig. S10A, B, D). Differently from E14 mES cells, parental R1 cells generate single rosettes that are more easily definable, allowing the measurement of two additional parameters: rosette area and the percentage of NESTIN + cells within the rosettes. Even here, Q tract length was found to correlate with rosette formation potential, as well as with lumen and rosette areas (Fig. S10E–H). In particular, the 0Q line generated fewer neural progenitors organized in rosettes and reduced rosette and lumen size, similar to unmodified R1 *Hdh*^*ex4–5*^ cells, and contrary to the 7Q cells where the wild-type rosette-forming condition was restored (Fig. S10E, I–K). Furthermore, interruption of the Q stretch with a non-synonymous CCG substitution, encoding proline in the 4th position of the polyQ tract (Q3PQ3 cells; Fig. S10A, C, D), reduces the amount of organized progenitors and generates smaller rosettes and lumens compared to R1 *Hdh*^*+/+*^ and 7Q cells (Fig. S10M–P). In contrast, a CAA synonymous substitution in the same codon (Fig. S10A, C, D) does not affect the rosette phenotype (Fig. S10M–P). We conclude that, also in conditions of overexpression, a 7Q tract is more effective in promoting rosette formation than 0Q, and that interrupting the purity of Q residues with only one proline disturbs rosette formation. In contrast, the known anti-apoptotic activity of HTT [58] was not affected in 0Q cells, which behaved as 7Q and *Hdh*^*+/+*^ cells (Fig. S10L), indicating that the anti-apoptotic activity of HTT does not reside within its polyQ tract.

To test whether small variations in the non-pathogenic polyQ size can have an impact on the phenotype at more advanced stages of neuronal differentiation, we subjected E14 mES cell-derived 0Q, 2Q, 4Q, and 7Q neural progenitors to pan-neuronal terminal differentiation until day 28 (Fig. 3A). Overall, terminally differentiated neuronal populations are more reminiscent of cortical cells regardless of Q length (Fig. S11A). We found that the level of the late-stage neuronal marker MAP2 in 7Q neurons is approximately 1.5-fold higher than in 0Q neurons (Fig. S11B, C). Moreover, an increase in the percentage of P27 and NEUN immunoreactive (post-mitotic) cells was found in 7Q compared to 0Q neurons (Fig. S11B, E, G). Fig. S11 also shows a positive correlation between the expression of late-stage neuronal markers (MAP2, P27, and NEUN) and the number of Qs, especially from 0 to 7Q, therefore providing further measurement of the ability of longer polyQ tract to affect biological parameters associated with neuronal differentiation (Fig. S11D, F, H).

All together, these experiments reveal that the length and purity of the HTT polyQ tract impacts aspects of neuronal cell biology in vitro.

### The HTT polyQ tract regulates pivotal genes linked to early-to-late neural development

**Fig. 4:**
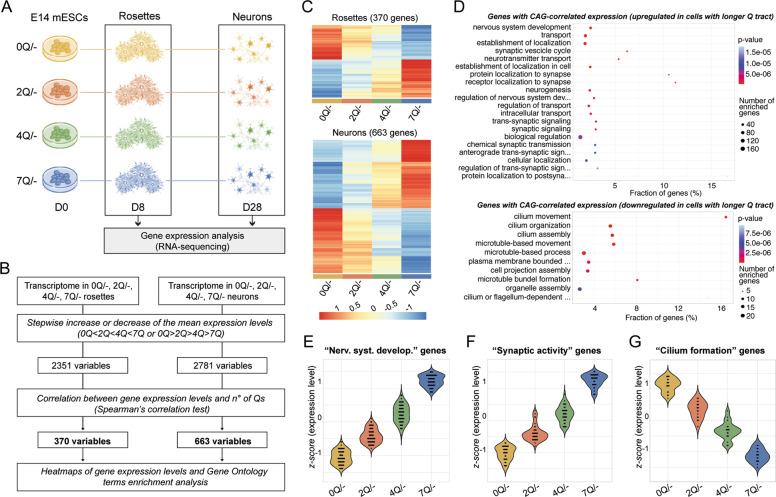
PolyQ length-sensitive influence in transcriptional programs governing in vitro neuronal differentiation. **A** Experimental scheme of gene expression analysis by RNA-seq at rosette and neuron stage among 0, 2, 4, and 7Q cell lines. **B** Schematic pipeline of whole transcriptomics-based analysis designed to identify genes whose expression increase or decrease stepwise (0Q > 2Q > 4Q > 7Q or 0Q < 2Q < 4Q < 7Q) and linearly (Spearman’s correlation) with CAG-length in 0, 2, 4, and 7Q transcriptomes. **C** Heatmaps of the expression levels for genes identified applying the pipeline depicted in **B**. The data was normalized and scaled. **D** GO dot plots of the top significant GO terms (ranked on their p-value from a classic Fisher test) of the genes, identified in **B**, which are upregulated (upper panel) or downregulated (bottom panel) in neurons with increasing number of Qs. For the full lists of DEGs see Supplementary dataset S21. **E**–**G** Violin plots of genes from **B** showing significant Q length-correlated expression selected for nervous system development (GO:0007399 term) (**E**), synaptic activity (GO:0099537, GO:0050808, GO:0035418 terms) (**F**) or cilium formation processes (GO:0060271 and GO:0044782 terms) (**G**) in 0Q, 2Q, 4Q, and 7Q neurons. Each dot represents the *z*-score value for each gene (*z*-score values were calculated by subtracting the population mean of expression levels from the sample mean of the raw expression levels and, then, dividing by the population standard deviation).

To explore the molecular changes that occur as a consequence of small length variations in the HTT polyQ tract which are reflected by the observed neural phenotypes (Fig. 3 and S11), we performed transcriptomic analyses of two cardinal stages of neuronal differentiation of 0, 2, 4, and 7Q knock-in E14 mES cell lines (Fig. 4A). Initially, we conducted a comprehensive investigation of the CAG length-driven molecular changes, considering the whole transcriptome of all lines at early (rosettes) and later (neurons) stage of cell differentiation (Fig. 4B). We found that 2351, for rosettes, and 2781 genes, for neurons, showed a stepwise increase (0Q > 2Q > 4Q > 7Q) or decrease (0Q < 2Q < 4Q < 7Q) in expression levels (Fig. 4B). Correlation analysis revealed that 370 genes, for rosettes, and 663 genes, for neurons, exhibit a significant correlation between the number of Qs and their expression levels (Fig. 4B, C). Notably, functional enrichment analyses (Fig. 4D) of genes showing a Q length-correlated expression at the later stage of differentiation revealed enriched gene ontology (GO) terms related to neuronal aspects, such as nervous system development (Fig. 4E), synaptic activity (Fig. 4F) and cilium formation (Fig. 4G), supporting the hypothesis that the non-pathogenic Q length in HTT affects parameters related to neuronal differentiation.

**Fig. 5:**
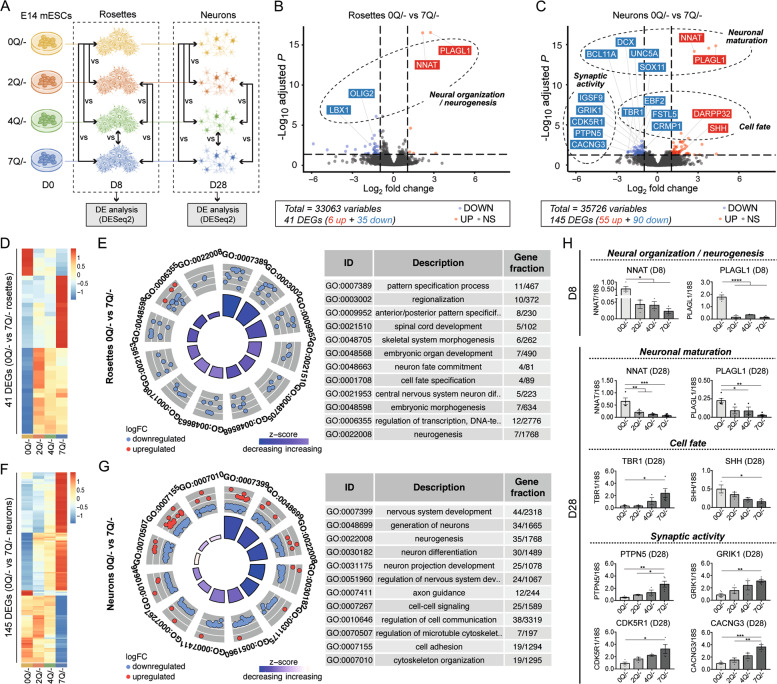
RNA-seq analyses reveal the HTT polyQ-related molecular pathways underlying different signatures in the early-to-late generation of neurons among 0, 2, 4 and 7Q cell lines. **A** Experimental scheme of gene expression analysis by RNAseq in which pairwise comparisons at rosette and neuron stage among 0, 2, 4, and 7Q (E14) cell lines were performed. **B**, **C** Volcano plots of DEGs between 0Q vs. 7Q at rosettes (**B**) and neuron (**C**) stage. Significant DEGs (adjusted *P*-value < 0.05) are labeled in red (for up-regulation with logFC > 1) or blue (for down-regulation with logFC < −1). The classes of genes subsequently validated by qPCR analysis are reported on the plot. **D** Heatmap of gene expression levels for significant DEGs between 0Q vs. 7Q rosettes (41 genes). The data was normalized and scaled. **E** Gene-ontology (GO) circle plot displaying the top significant (ranked on their *P*-value) non-redundant GO terms of the DEGs between rosettes 0Q vs. 7Q. Within each selected GO term the outer chart shows the distribution of the logFC of the individual assigned genes from higher (outer layer) to lower (inner layer). Genes are represented as red (upregulated) and blue (downregulated) circles. The height of the inner bars represents the *P*-value of the GO term whereas its color indicates the *z*-score (see “Methods” section). **F** Heatmap of gene expression levels for significant DEGs between 0Q vs. 7Q neurons (145 genes). The data was normalized and scaled. **G** GO circle plot displaying the top significant (ranked on their *P*-value) non-redundant GO terms of the DEGs between neurons 0Q vs. 7Q. For the details of the plot see **E**. **H** qPCR validation analyses of significant DEGs between 0Q vs. 7Q cell lines at rosette (D8) and neuron (D28) stage. Bar plots display the mean values ± SEM from *n* ≥ 3 biological replicates; *P < 0.05, **P < 0.01, ***P < 0.001, ****P < 0.0001, one-way ANOVA test followed by Tukey. Values of the logFCs are scaled. For the full lists of DEGs see Supplementary dataset S21.

We then identified the main actors involved in Q length-related phenotypes by performing pairwise comparisons among all the cell lines at two time-points, rosettes and neurons (Fig. 5A and S12), with a specific focus between 0Q and 7Q lines. We detected differentially expressed genes (DEGs) between 0Q and 7Q genotypes at both rosette and neuron stages, with a higher number of DEGs in the latter (Fig. 5B, C). A total of 41 DEGs in 0Q versus 7Q rosettes are enriched in GO terms related to neuro-development (Fig. 5B, D, E and Supplementary dataset S21). Among these genes, we found *NNAT, PLAGL1* (also known as *ZAC1*), *LBX1* and *OLIG2*, which are implicated in the organization of neuroepithelial progenitors during neurogenesis (Fig. 5B) [59–64]. We confirmed by qPCR that 0Q neural progenitors show abnormally elevated expression of neurogenic factors *NNAT* and *PLAGL1* (Fig. 5H), whose dysregulation may affect the ability of 0Q cells to correctly generate neural rosettes.

When 0Q and 7Q cultures were compared at the more mature neuronal stage, 145 DEGs were detected. Among them, we found in the 0Q line a deregulation of genes implicated in neuronal maturation, cell fate, and synaptic activity (Fig. 5C, F, G and Supplementary dataset S21), reflecting the increased capacity of the 7Q line of acquiring more mature neuronal signatures. Among neuronal maturation DEGs, we confirmed by qPCR that *NNAT* and *PLAGL1* were elevated in 0Q compared to 7Q neurons (Fig. 5H) with possible consequences for terminally differentiated 0Q neurons [62, 63]. We also found increased expression of the typical dorsal marker *TBR1* (Fig. 5H) (as well as of other cortical markers such as *EBF2*, *FSTL5*, and *CRPM1*; Fig. 5C) and reduced expression of the ventral marker *SHH* (Fig. 5H) in 7Q neurons. Finally, we validated a third class of DEGs implicated in synapse activity and dendrite outgrowth (such as *CDK5R1*, *PTPN5*, *GRIK1*, and *CACNG3*) whose expression was found to be increased in 7Q compared to 0Q neurons (Fig. 5H).

Collectively, these results show that the HTT polyQ tract is biologically active and seems to orchestrate key factors of in vitro neural organization, cell fate, and neuronal maturation processes.

## Discussion

In this study, by exploring vertebrate genomes, we provide the strongest evidence to date that the CAG repeats in the *HTT* gene are the product of purifying selection against deleterious mutations. Purifying natural selection is a hallmark of functional biological sequences and the non-pathogenic CAG-polyglutamine repeats in HTT must confer advantageous traits to the protein. Consistent with this, we discovered that the HTT non-pathogenic polyQ tract has neuronal activities. In line with our finding, previous studies have shown that Q deletion in mice causes cognitive deficits in specific tests [65]. Moreover, studies conducted in humans, suggest that an increase in the number of CAG repeats in *HTT*—below the disease threshold—confers positive changes in human brain structure and general intelligence [22–24] as well as improvements in cognitive performance [25].

In our study we found a positive correlation between the length of the non-pathogenic polyQ tract and phenotypes, demonstrating that very small variations in the number of CAG repeats are sufficient to mediate the transcriptional response of differentiating neuronal cells. Our transcriptome analyses propose that polyQ tract length influences neural phenotypes by modulating the expression of genes involved in neural organization, neuronal identity, synapse maturation, and cilium formation. Of note, we found that an elongating CAG tract positively affects genes involved in vesicle trafficking as well as in synapse architecture and activity, while it negatively affects genes involved in ciliogenesis. These findings consistently emphasize some of the known physiological functions of HTT [8, 9] and corroborate the hypothesis that longer physiological polyQ tract could possibly generate more mature, more connected and more functional neurons. Curiously, many of the processes—that we found to be regulated by the polyQ tract in our in vitro models—are the same ones on which natural selection usually operates to promote the evolution of neural circuits [66]. Therefore, we hypothesize that evolution has selected for longer CAG HTT stretches since they carry advanced neuronal activities.

Overall, our findings demonstrate that the HTT polyQ tract is functionally active in our neural cell assays and that small size fluctuations in the number of Qs influence its biological activity. The CAG repeat in the *HTT* gene may therefore have been selected throughout evolution to act as a genetic modulator of brain development and function [15].

Our study provides experimental evidence of the neural activity of the HTT physiological polyQ tract, supporting the hypothesis that simple sequence repeats, as CAG repeats, play a fundamental role in evolution by producing the variability needed to enhance changes in neuronal/brain development [15, 67, 68]. Previous works suggest that CAG-encoded polyQ tracts are enriched in genes with neuronal functions [53] while polyQ diseases always exhibit elements of neurological dysfunction [69], consistent with the hypothesis that these three-nucleotide repeats might indeed be physiologically relevant.

HTT is highly expressed in brain and testis and it is also found in other non-neuronal tissues [70, 71]. The CAG tract length may therefore also affect activities of HTT in other tissues. For example, in vivo studies have shown that the protein is implicated in spermatogenesis by regulating translation and DNA packaging in the testis [72]. Whether natural selection is operating more broadly, not exclusively on CAG-dependent neuronal phenotypes, remains to be elucidated.

The molecular machinery responsible for the elongation of the HTT CAG tract is unknown. Evidence that a pure CAG tract leads to instability and expansion of the tract, while CAA interruptions leads to a more stable tract [7] allows us to hypothesize that this mechanism may also occur during the expansion observed during evolution. There may also be *trans*-acting modifiers, such as genes linked to the control of DNA replication and repair [73] that have co-evolved with HTT, allowing for the expansion of the CAG tract in mammals especially in humans, as seen in the disease [74]. Hence, the pathological CAG repeat found in patients may represent the side effect of an evolutionary path that aims at maintaining the purity of an elongating and beneficial CAG repeat in the human gene. In this complex scenario, human HTT Q repeat lengths may reflect a delicate balance between the evolutionary advantages of having a pure and longer tract and the detrimental effects of an extreme expansion.

Our findings may lead to a paradigm shift in our view of a rare genetically dominant adult-onset neurological disease whose underlying genetic trait has not been selected against. Historically, it has been assumed that the preservation of an elongated repetitive tract in the human state was due to a simple length-dependent mutational bias towards longer alleles [11]. Our study, on the other hand, advocates that the HTT polyQ tract has been selected throughout evolution, possibly to pursue its functional neural purpose.

## Supplementary information

Figure 1 (TIFF file in CMYK format with high resolution of 300 dpi)

Figure 2 (TIFF file in CMYK format with high resolution of 300 dpi)

Figure 3 (TIFF file in CMYK format with high resolution of 300 dpi)

Figure 4 (TIFF file in CMYK format with high resolution of 300 dpi)

Figure 5 (TIFF file in CMYK format with high resolution of 300 dpi)

Supplementary information

PRE Authorship+form_spring nature - pre-acc journals

Dataset 1

Dataset 2

Dataset 3

Dataset 4

Dataset 5

Dataset 6

Dataset 7

Dataset 8

Dataset 9

Dataset 10

Dataset 11

Dataset 12

Dataset 13

Dataset 14

Dataset 15

Dataset 16

Dataset 17

Dataset 18

Dataset 19

Dataset 20

Dataset 21

Dataset 22

Dataset 23

Dataset 25

Dataset 26

## Data Availability

The authors declare that all data supporting the findings of this study are available within the paper in the main text or the supplementary materials. Bulk RNA-seq data have been deposited in the ArrayExpress database at EMBL-EBI (https://www.ebi.ac.uk/arrayexpress/) under accession no. E-MTAB-9378.
