# Supplementary figures and images for: The evolutionary history of the polyQ tract in huntingtin sheds light on its functional pro-neural activities
Source: Cell Death Differ. 2022 Jan 1;29(2):293–305. doi: 10.1038/s41418-021-00914-9 (PMC8817008; doi:10.1038/s41418-021-00914-9)

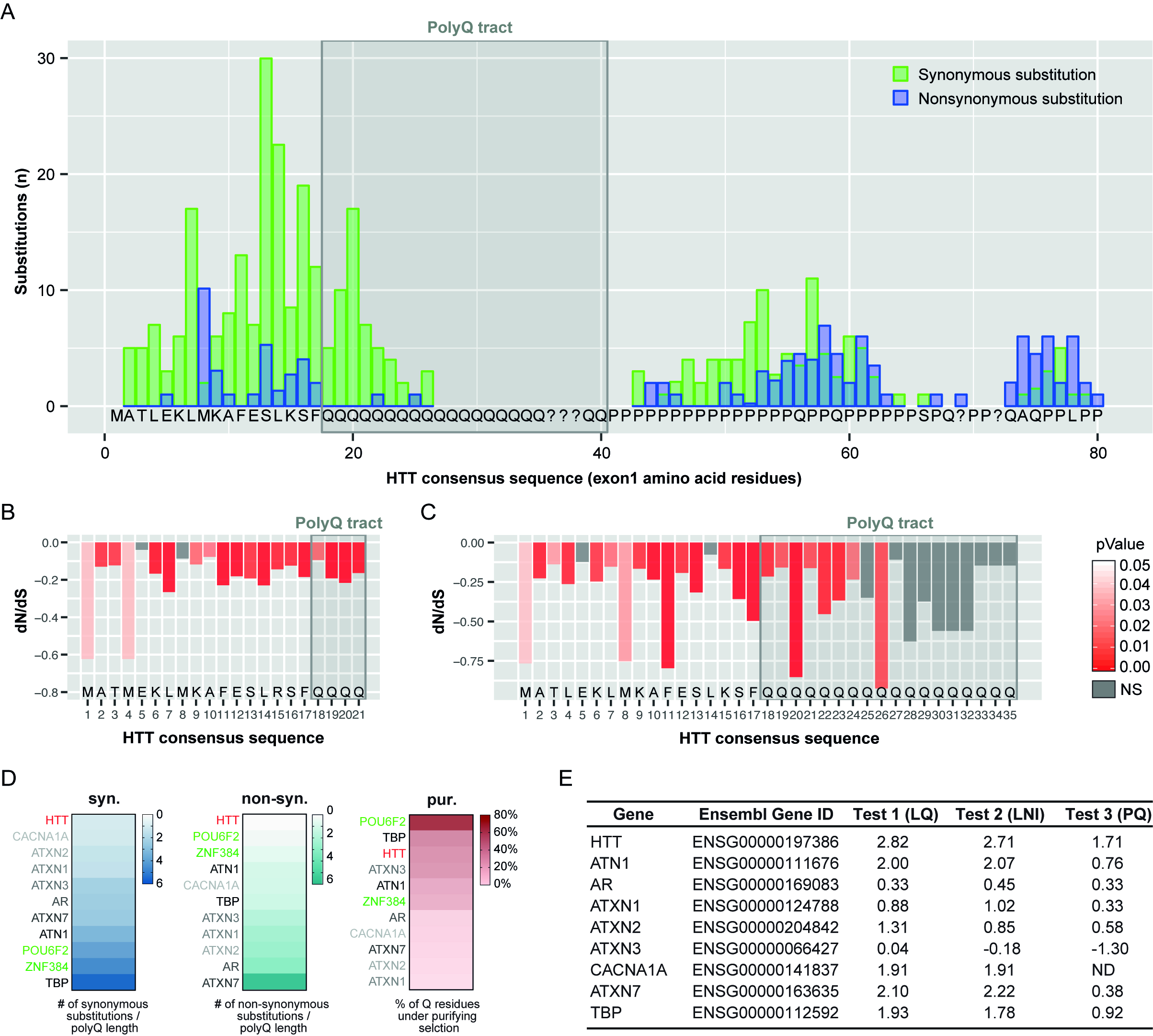

Supplement: Supplementary file 1 — Figure 1 (TIFF file in CMYK format with high resolution of 300 dpi) [file 41418_2021_914_MOESM1_ESM.tif]

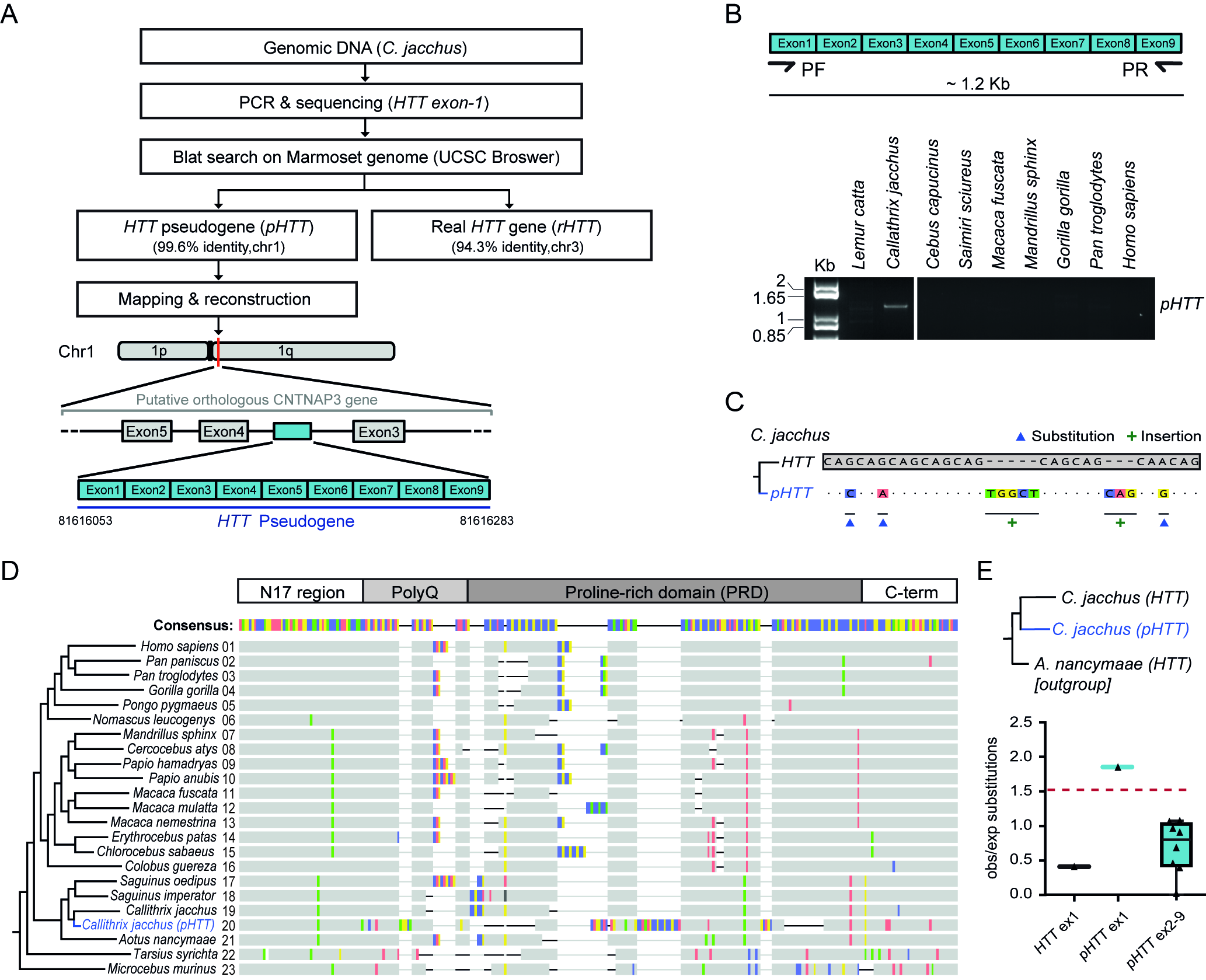

Supplement: Supplementary file 2 — Figure 2 (TIFF file in CMYK format with high resolution of 300 dpi) [file 41418_2021_914_MOESM2_ESM.tif]

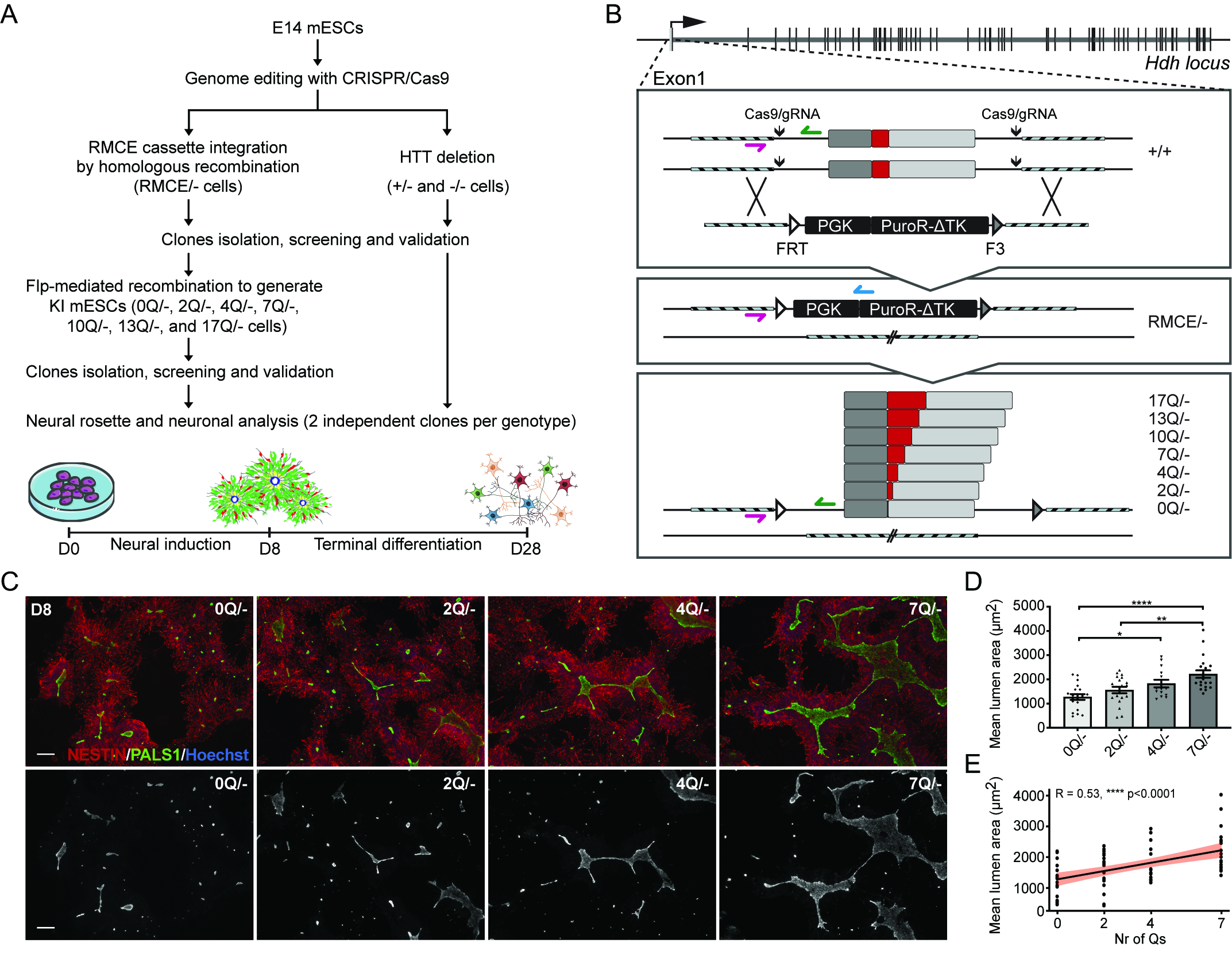

Supplement: Supplementary file 3 — Figure 3 (TIFF file in CMYK format with high resolution of 300 dpi) [file 41418_2021_914_MOESM3_ESM.tif]

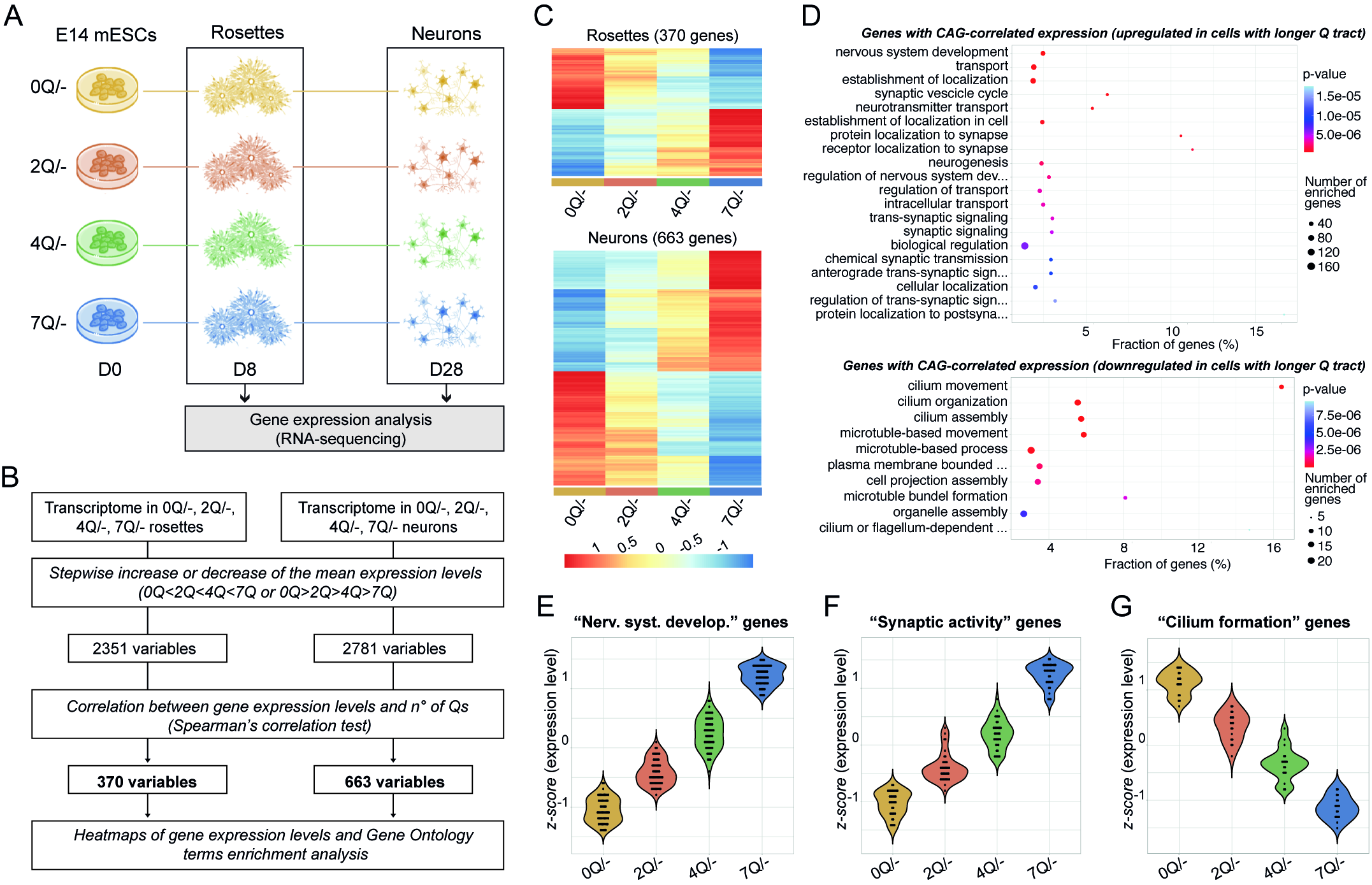

Supplement: Supplementary file 4 — Figure 4 (TIFF file in CMYK format with high resolution of 300 dpi) [file 41418_2021_914_MOESM4_ESM.tif]

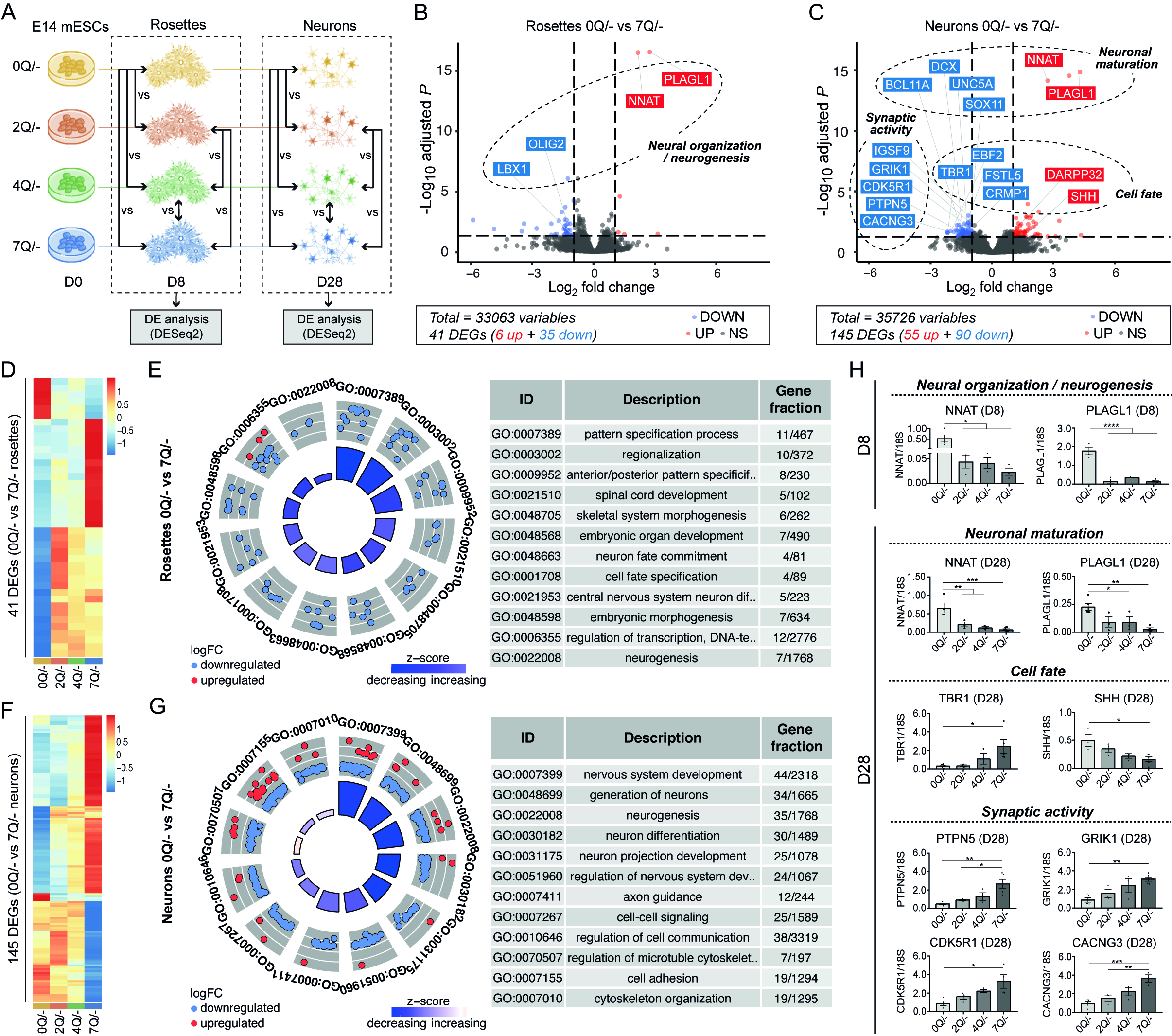

Supplement: Supplementary file 5 — Figure 5 (TIFF file in CMYK format with high resolution of 300 dpi) [file 41418_2021_914_MOESM5_ESM.tif]
