# Supplementary material for: The evolutionary history of the polyQ tract in huntingtin sheds light on its functional pro-neural activities
Source: Cell Death Differ. 2022 Jan 1;29(2):293–305. doi: 10.1038/s41418-021-00914-9 (PMC8817008; doi:10.1038/s41418-021-00914-9)
Supplement: Supplementary file 6 — Supplementary information [file 41418_2021_914_MOESM6_ESM.doc]

**Supplementary information for**

**The evolutionary history of the polyQ tract in huntingtin sheds light on its functional pro-neural activities**

Raffaele Iennaco, Giulio Formenti, Camilla Trovesi, Riccardo Lorenzo Rossi, Chiara Zuccato, Tiziana Lischetti, Vittoria Dickinson Bocchi, Andrea Scolz, Cristina Martínez-Labarga, Olga Rickards, Michela Pacifico, Angelica Crottini, Anders Pape Møller, Richard Z. Chen, Thomas F. Vogt, Giulio Pavesi, David Stephen Horner, Nicola Saino and Elena Cattaneo

**This file includes:**

Figures S1 to S12

Legends for Datasets S1 to S26

**Other supplementary information for this manuscript include the following:**

Datasets S1 to S26 (separate files)

Figure S1: Multiple alignment of *HTT* exon1.

Alignment was performed at the codon level and the figure displays translated sequences. Two CAG-encoded Qs are present in Echinodermata (*Strongylocentrotus purpuratus*), Hemichordata (*Saccoglossus kowalevskii*), Cephalochordata (*Branchiostoma lanceolatum* and *Branchiostoma floridae*) and up to Chondrichthyes (*Callorhinchus milii*). The transition to 4Q occurs in bony fishes (e. g., *Danio rerio*) that, together with all birds and the majority of the amphibians and reptiles tested, invariably showed 4Q. The transition between ‘lower’ vertebrates and mammals, which show Q≥4, coincides with the appearance of a polyA/polyP (CCN/GCN) region immediately following the polyQ in most analysed Squamata, including members of the families Gekkonidae, Scincidae, Phyllodactylidae, Dactyloidae and Lamprophiidae, but not observed in the analysed members of the families Lacertidae and Colubridae. Mammals show wide polyQ length variation, from 4Q found in guinea pigs-related clades (infraorder Hytricognathi) and pangolins (*Manis javanica*, order Pholidota) to 35Q in healthy humans. While polyQ mean length is shorter in mammals other than humans, several species, including cows (*Bos taurus*, 15Q) and pigs (*Sus scrofa*, 18Q), show polyQ length within the human range. Noteworthy, the polyQ region is not interrupted by other amino acids, except in two extremely rare cases in which a histidine appears in the middle of the tract (*Erythrocebus patas* and *Procavia capensis*). Whenever possible, more than one sample per species has been sequenced (as listed in Supplementary dataset S1).


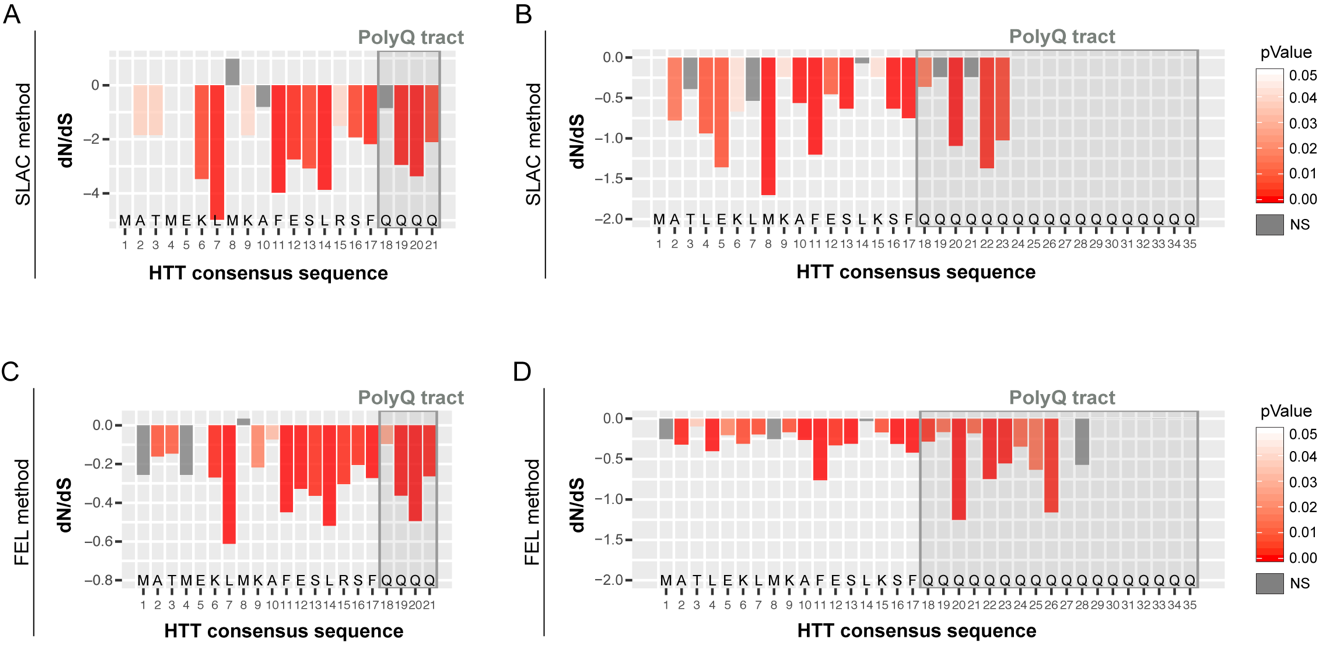


**Figure S2:** **Effects of natural selection on *HTT* exon1 determined according to FEL and SLAC tests.**

The figure shows the results of the dN/dS ratio determined by alternative computations: (A) SLAC test and (C) FEL method on the MSA subsets (n = 84 species) with 4 glutamine-encoding codons respectively, and (B) SLAC test and (D) FEL method on the mammals MSA subsets (n = 74 species) where the number of glutamine-encoding codons is variable (Q≥4). Direction of peaks indicates kind of selection (downward = purifying/negative; upward = diversifying/positive); peaks height indicates dN/dS values and peak colors (shades of red) show significance level. Consensus HTT N-terminal sequence for each alignment is shown for reference. PolyQ tracts shaded in the grey boxed area.


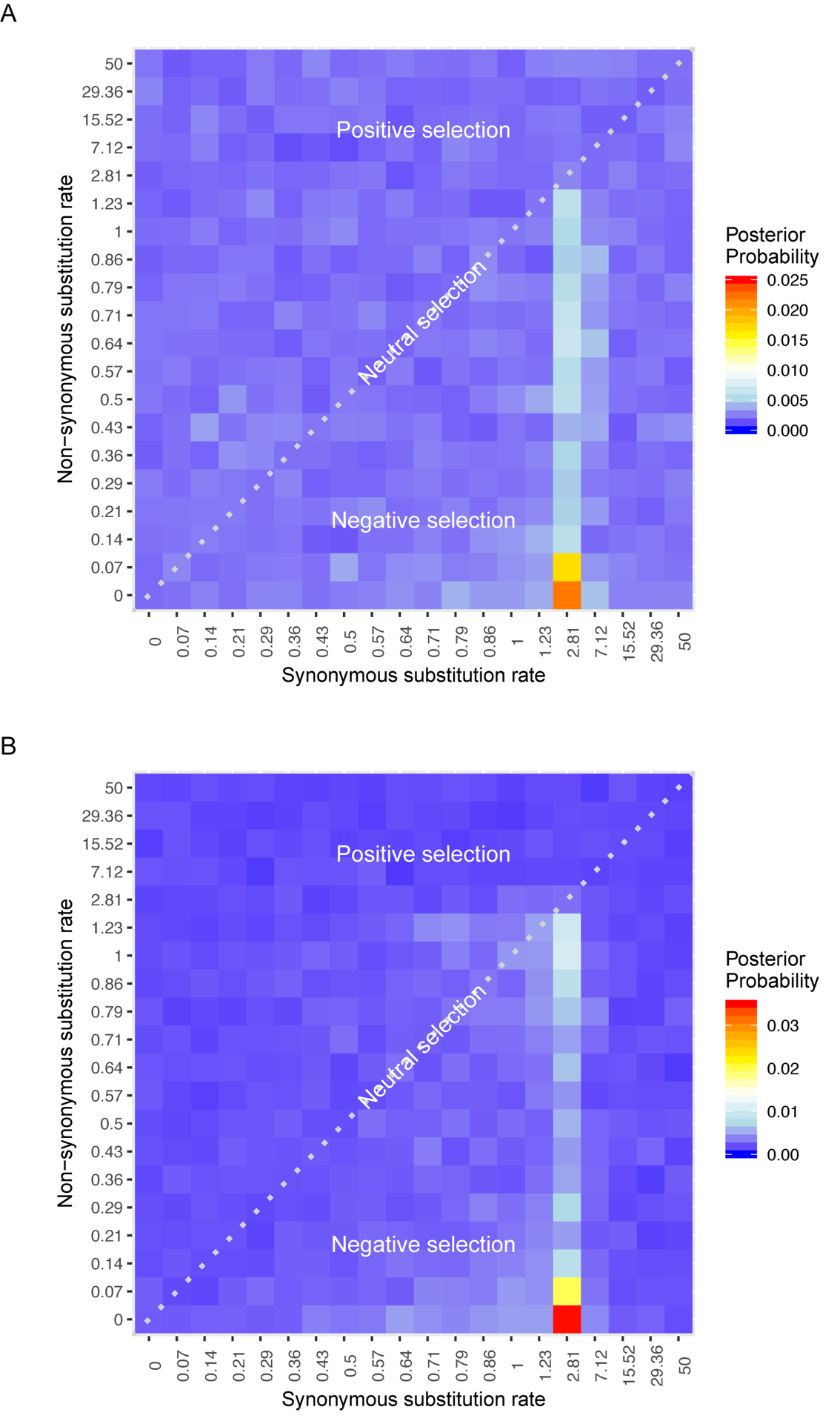


**Figure S3:** **Alignment-wide distribution of substitution rates probability.**

Probability distribution of pervasive selection as estimated by FUBAR method determined over a discretized grid of 20 synonymous and non-synonymous rates values for a total of 400 sampled grid points of which 20 represent neutral evolution (across the diagonal dotted line, where syn. rate = non-syn. rate), 190 positive selection (upper left triangle), and 190 negative selection (lower right triangle). The color gradient is relative to the posterior weight in each grid point, from lower (blue) to higher (red) probability. Distribution (A) for non-mammalian species with 4Q and (B) for mammalian species with polyQ of variable length (Q≥4).

**
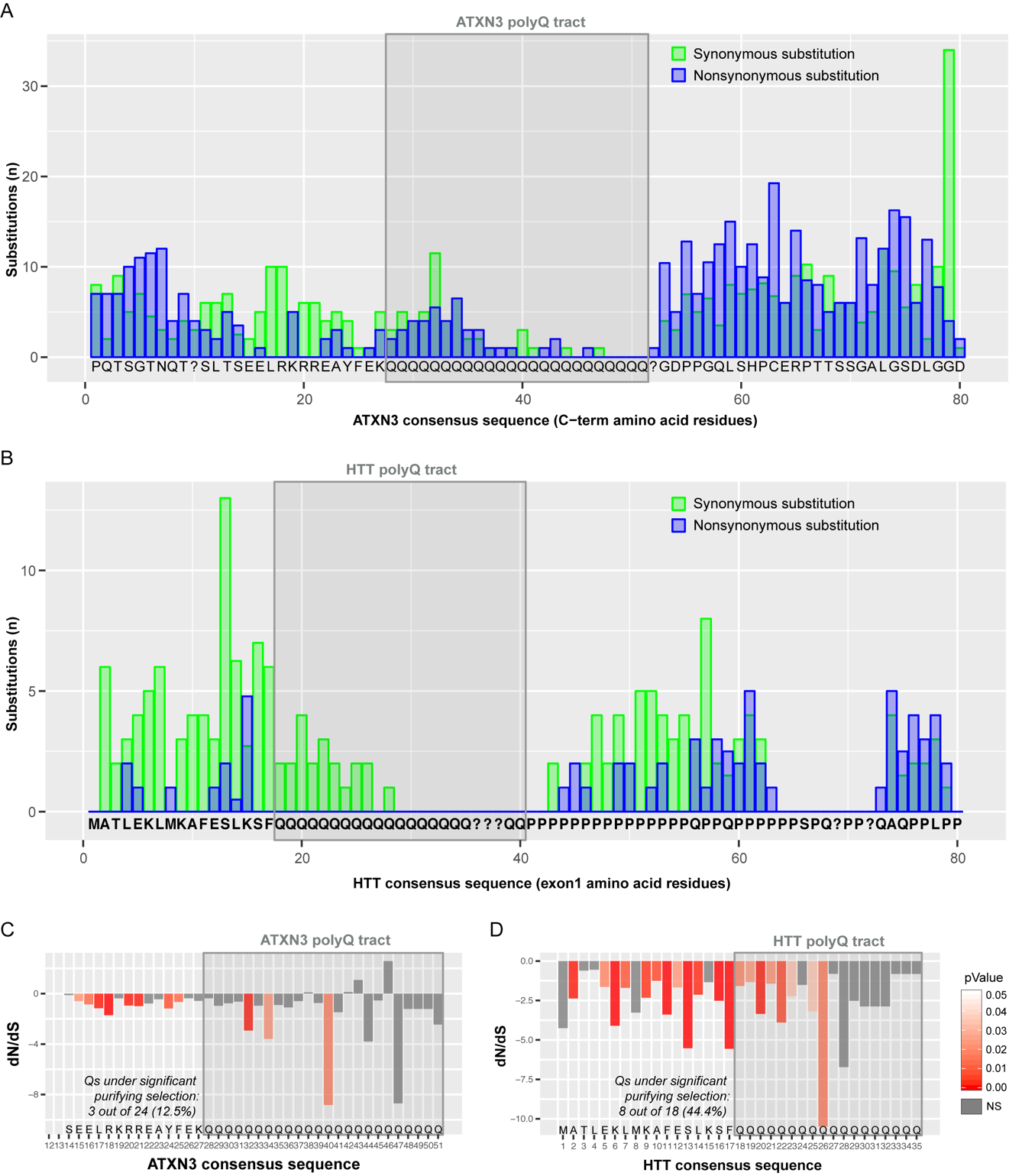
**

**Figure S4:** **Comparative analysis between HTT and ATXN3 polyQ tract conservation.**

**A** Synonymous and non-synonymous substitutions counted with the codon-based maximum likelihood method SLAC on the multiple alignment of 53 unique sequences of Ataxin-3 gene (*ATXN3*); synonymous (green) and non-synonymous (blue) substitution counts are shown for each codon (consensus sequence in the plot). The grey shaded box indicates polyQ stretch. **B** Synonymous and non-synonymous substitutions counted with SLAC method for an *HTT* exon1 alignment of the same 53 taxa used in the *ATXN3* alignment. The grey shaded box indicates polyQ stretch. **C** Purifying selection determined by FUBAR method for the *ATXN3* alignment. Consensus sequence of the C terminal stretch of ATXN3 containing the polyQ stretch is shown. **D** Purifying selection determined by the FUBAR method for the *HTT* exon1 alignment. Consensus sequence of the N terminal stretch of HTT containing the polyQ stretch is shown. A time-tree of the 53 taxa was used as backbone for calculations. The grey shaded boxes indicate polyQ tracts. Histograms show dN/dS values (where negative values indicate purifying selection). Colour gradients indicate p-values (darker red = more significant), grey bars show non-significant results.

**
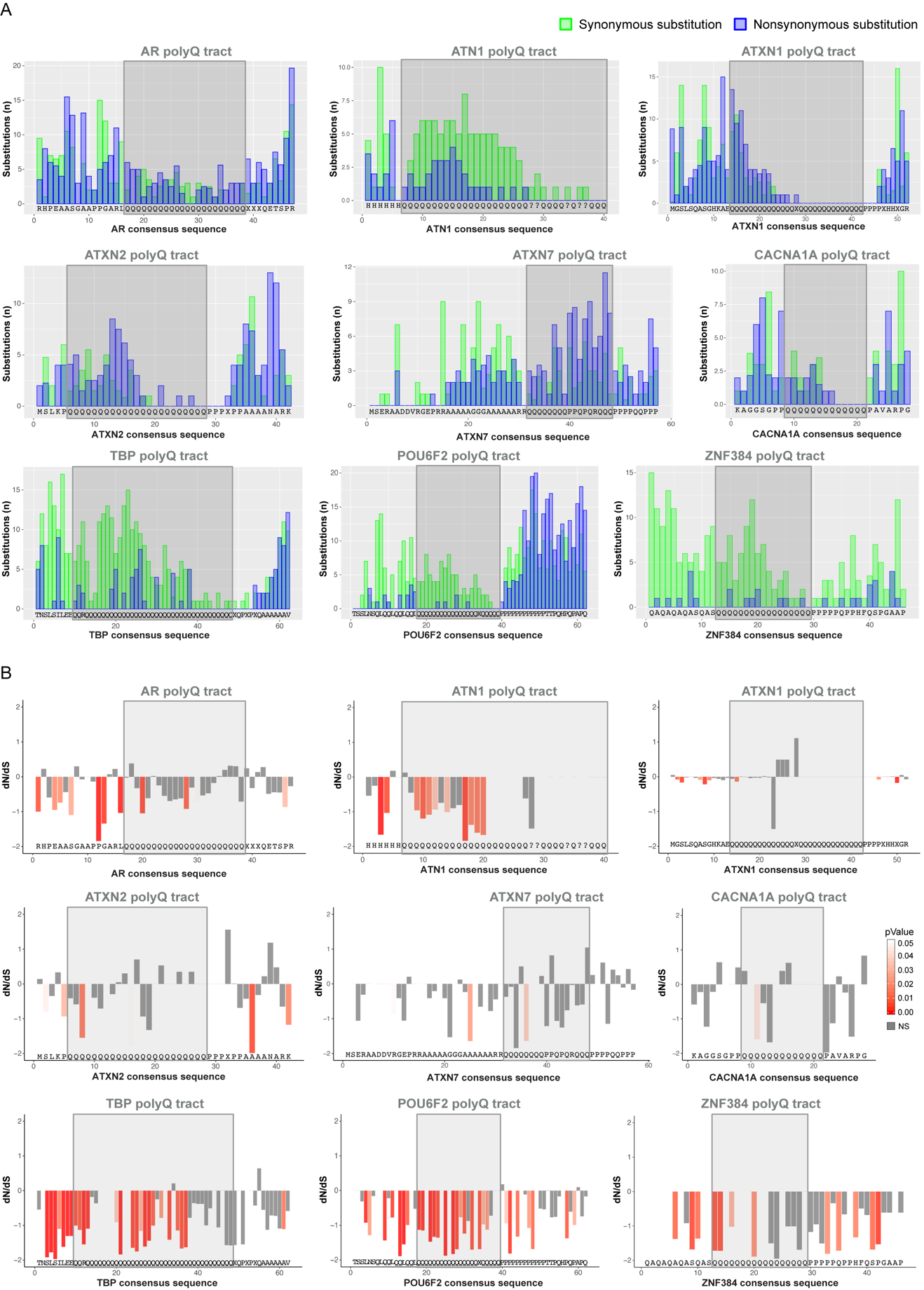
**

**Figure S5:** **Analysis of the polyQ tract conservation in other polyQ-containing proteins.**

**A** Synonymous and non-synonymous substitutions counted with the codon-based maximum likelihood method SLAC on the multiple alignments of *n* unique sequences for each gene, where *n* and gene are: 84 for *AR*; 85 for *ATN1*; 77 for *ATXN1*; 48 for *ATXN2*; 42 for *ATXN7*; 46 for *CACNA1A*; 94 for *TBP*; 74 for *POU6FA*; 81 for *ZNF384*. Synonymous (green) and non-synonymous (blue) substitution counts are shown for each codon (consensus sequence in the plot). The grey shaded box indicates the polyQ stretch. **B** Purifying selection determined by SLAC method for the same alignments, a time-tree obtained for each group of taxa in all alignments was used as backbone for calculations. Consensus sequence of residues surrounding the polyQ stretch is shown. The grey shaded boxes indicate polyQ tracts. Histograms show dN/dS values (where negative values indicate purifying selection). Colour gradients indicate p-values (darker red = more significant), grey bars show non-significant results.


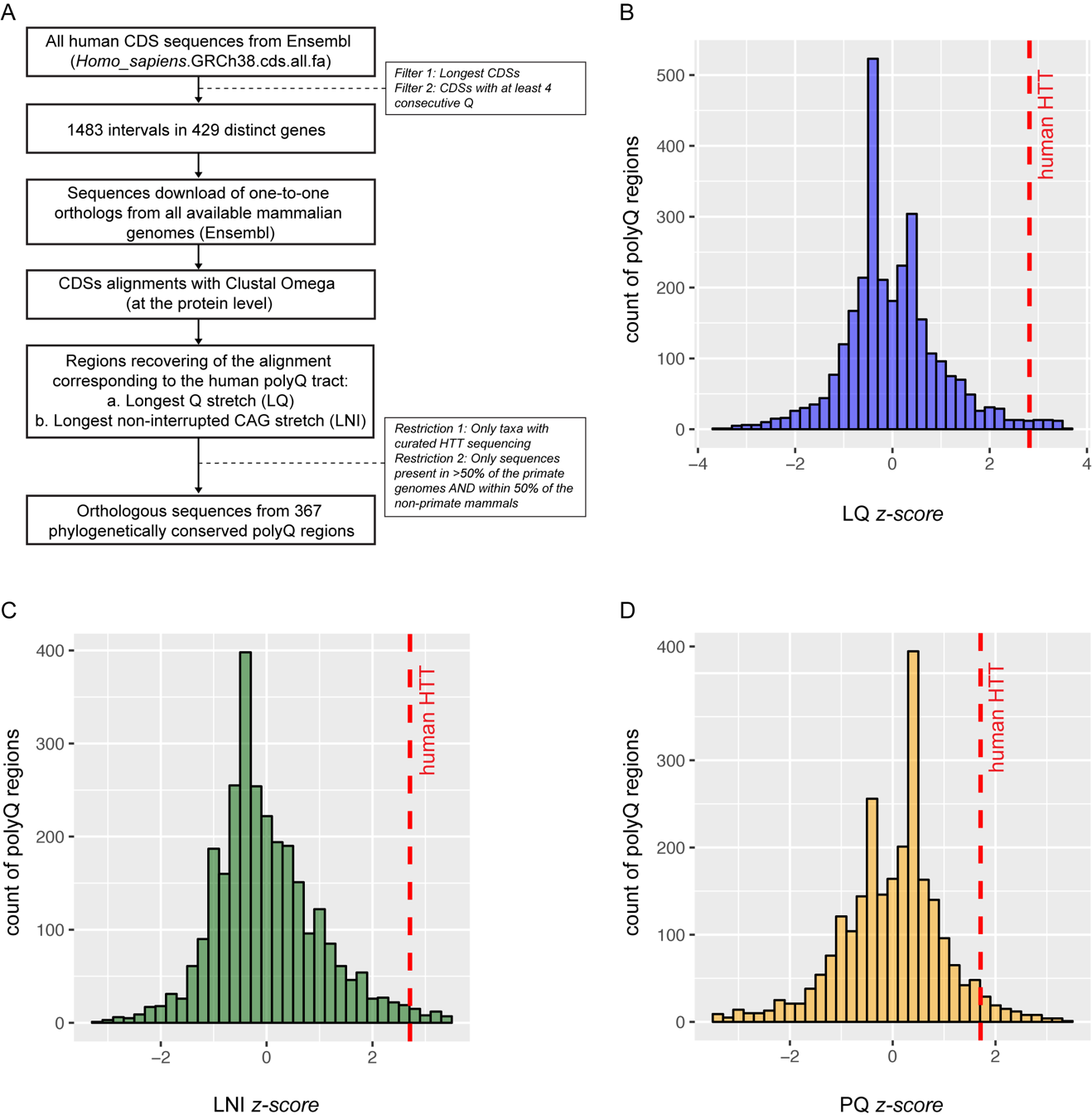


Figure S6: Comparative analysis of the polyQ proteins in mammalian genome.

A Pipeline to retrieve polyQ regions that are conserved and variable along primates and other mammals. B Test 1: distribution of polyQ regions in terms of longest Q stretch (LQ), where each value represents the LQ *z-score* (difference between the observed LQ and the mean LQ for orthologous tracts, divided by the standard deviation of LQ for orthologous tracts). C Test 2: distribution of polyQ regions in terms of longest non-interrupted CAG interval (LNI) where each value represents the LQ *z-score* (difference between the observed LNI and the mean LNI for orthologous tracts, divided by the standard deviation of LNI for orthologous tracts). D Test 3: distribution of polyQ regions in terms of the polyQ purity (i.e. CAG/CAA proportion) (PQ), where each value represents the PQ *z-score* (difference between the observed PQ and the mean PQ for orthologous tracts, divided by the standard deviation of PQ for orthologous tracts). Dashed lines represent the obtained values of the human HTT polyQ region in each test. The full lists of polyQ proteins with atypical LQ, LNI and PQ z-score values are reported in Supplementary dataset S18.

**
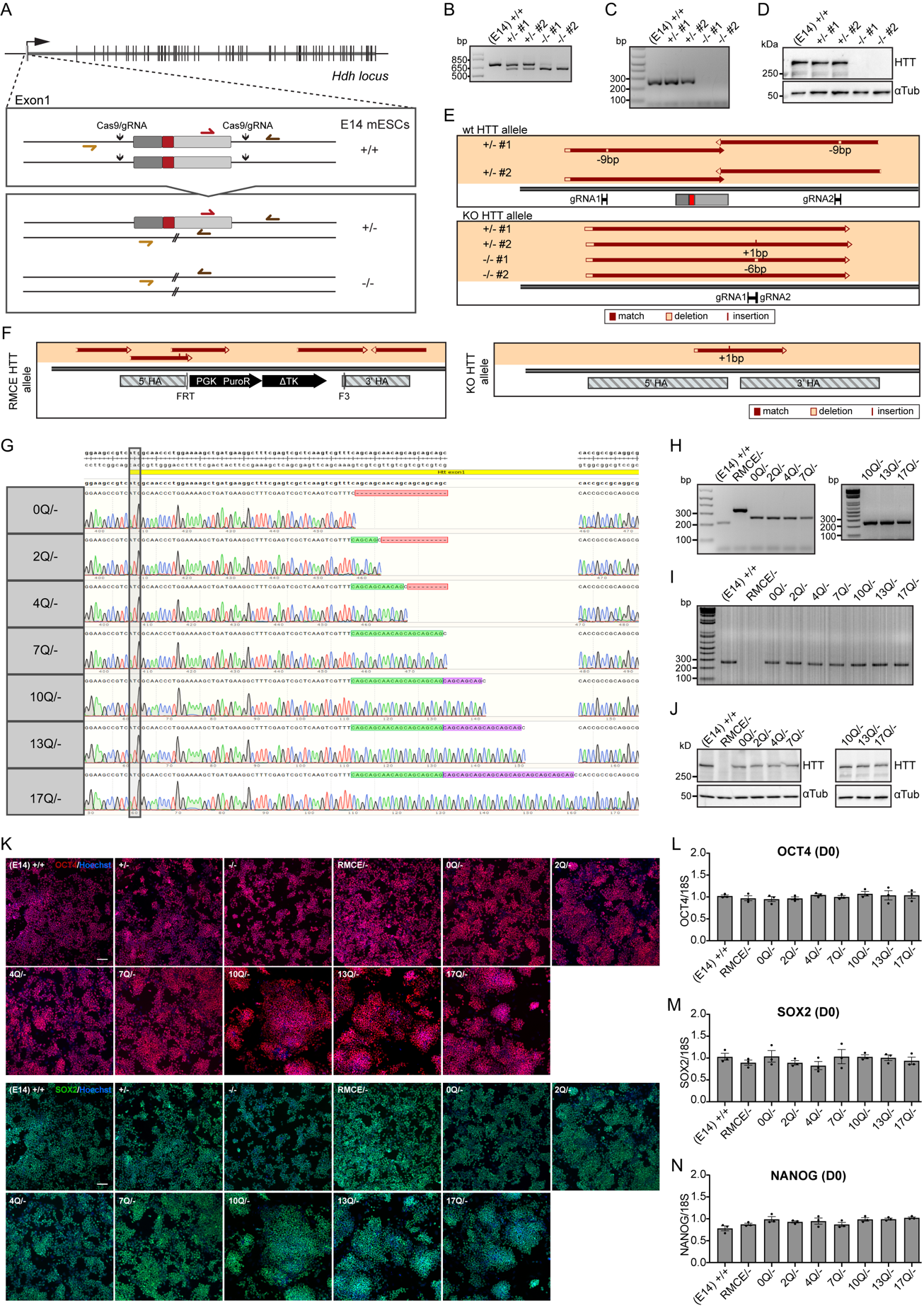
**

**Figure S7:** **HTT deletion in E14 mESCs and quality control on knock-out and knock-in cell lines.**

**A** Genome editing strategy used to produce knock-out mESCs. HTT deletion was achieved by CRISPR/Cas9 with two gRNAs, cutting upstream and downstream the exon1 coding region, giving rise to heterozygous (+/-) and homozygous (-/-) cell lines. **B-E** Quality control on editing and HTT expression. (B) PCR assay to verify HTT deletion was performed with the three oligos depicted in panel A (orange, red and brown). In heterozygous clones both the wt (677bp) and KO (595bp) bands are detectable. (C) RT-PCR amplifying exon3-6 and (D) MAB-2166 (anti-HTT) Western blot to visualize HTT expression in +/+, +/- and -/- mESCs. (E) Upper box, Sanger sequencing alignments of HTT wt alleles amplified by PCR from the two +/- clones. HTT Exon1 (grey/red box) and gRNA1 and 2 sequences (|-|) are reported on the wt HTT locus sequence (black double line). Lower box, Sanger sequencing alignments of KO HTT alleles amplified by PCR from the two +/- and the two KO clones; black double line represents the expected KO sequence, bearing the precise deletion of the HTT genomic region between gRNA1 and 2 sequences (|-|). **F** Left box, Sanger sequencing alignments of RMCE HTT allele amplified by PCR from the RMCE/- cell line. 5’ and 3’ homology arms (HA) and RMCE cassette (from FRT to F3 recombination sites) are reported on the expected RMCE HTT locus (black double line). Right box, Sanger sequencing alignment of KO HTT allele amplified by PCR from the RMCE/- clone; black double line represents the expected KO sequence, bearing the precise deletion of the HTT genomic region between 5’ and 3’ HA. **G** Sanger sequencing alignments of HTT knock-in exons1 amplified by PCR from 0Q/-, 2Q/-, 4Q/-, 7Q/-, 10Q/-, 13Q/-, and 17Q/- cell lines. (*H*-*J*) Quality control on editing and HTT expression. **H** PCR assay to verify RMCE integration and exchange was performed with the three oligos depicted in **Fig. 3B** (pink, green and blue arrows), designed to amplify a region spanning FRT recombination site. The expected amplification bands were 207bp before RMCE integration, 337bp after RMCE integration and 255bp upon RMCE cassette exchange. **I-J** (I) RT-PCR amplifying exon3-6 and (J) D7F7 (anti-HTT) Western blot to detect HTT expression in self-renewal condition. **K** Representative images of +/+, +/-, -/-, RMCE/, 0Q/-, 2Q/-, 4Q/-, 7Q/-, 10Q/-, 13Q/-, and 17Q/- ES cells stained for OCT4 and SOX2 in self-renewal. The scale bars correspond to 50 μm. **L-N** qPCR for *OCT4*, *SOX2*, *NANOG* in +/+, RMCE/-, 0Q/-, 2Q/-, 4Q/-, 7Q/-, 10Q/-, 13Q/-, and 17Q/- ES cells at day 0 (n = 3 biological experiments, data are represented as mean ± SEM). gRNAs and oligos sequences are reported in **Supplementary dataset S23**. Raw gel images are reported in **Supplementary dataset S24**.

**
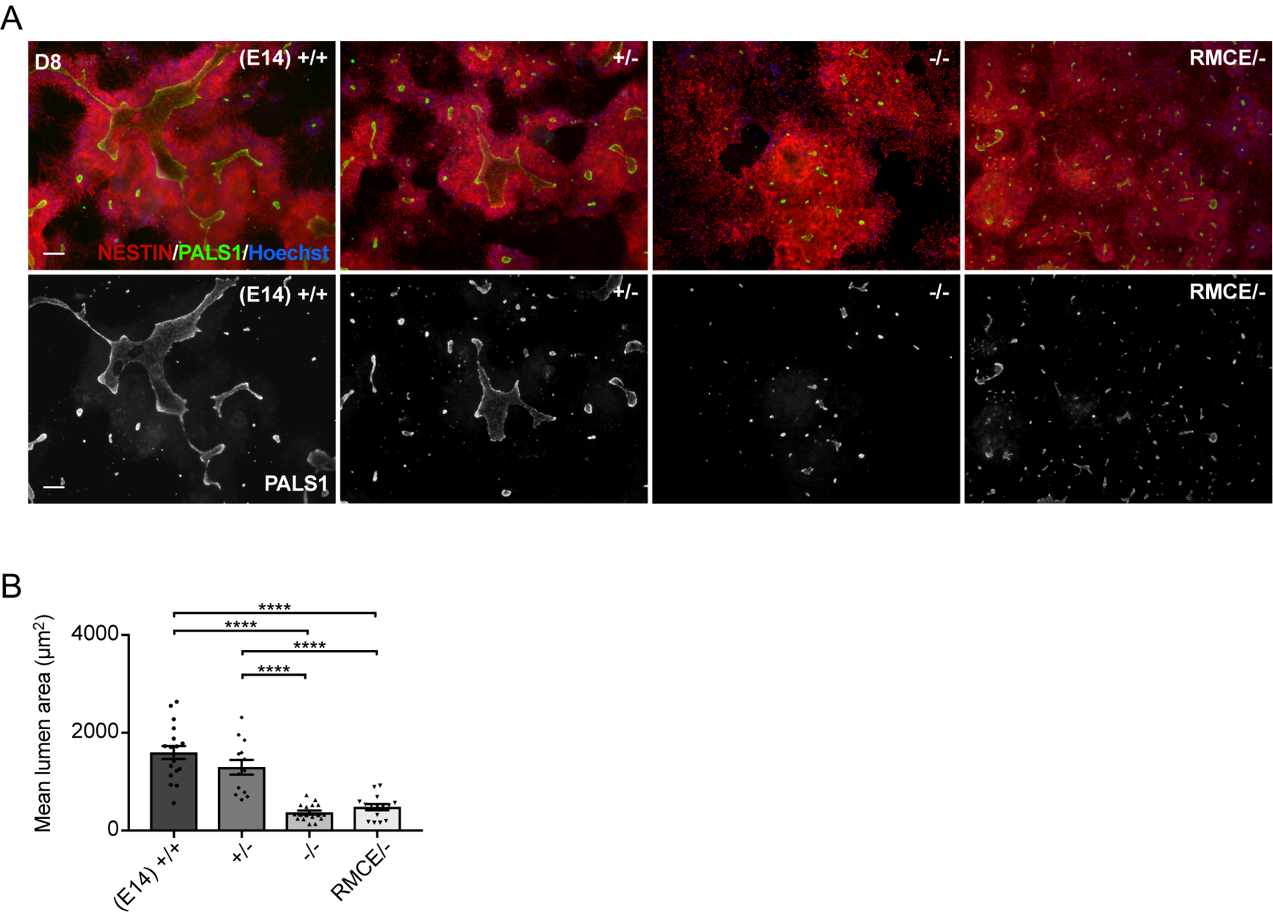
**

**Figure S8: Effects of normal HTT deletion in a (E14) ES cell-derived neural rosette formation assay.**

**A** Representative images of rosette/lumen phenotype in E14 wild-type (+/+), heterozygous (+/-) and homozygous (-/-; RMCE/-) HTT knock-out control cell lines stained for PALS1 and NESTIN at day 8 after neural induction. **B** Mean lumen area (μm^2^) of E14 +/+, +/-, -/- and RMCE/- cell cultures automatically quantified with CellProfiler. Data are expressed as mean ± SEM from n≥5 independent neural differentiation experiments. Each dot represents the mean lumen area per well testing a pool of 2 clones for each edited cell line (see **Supplementary dataset S25** for raw data and for the exact number of biological replicates per each genotype). **** P<0.0001, one-way ANOVA test followed by Tukey. All images were automatically acquired by IN Cell Analyzer 6000. The scale bars correspond to 50 μm. ­­­


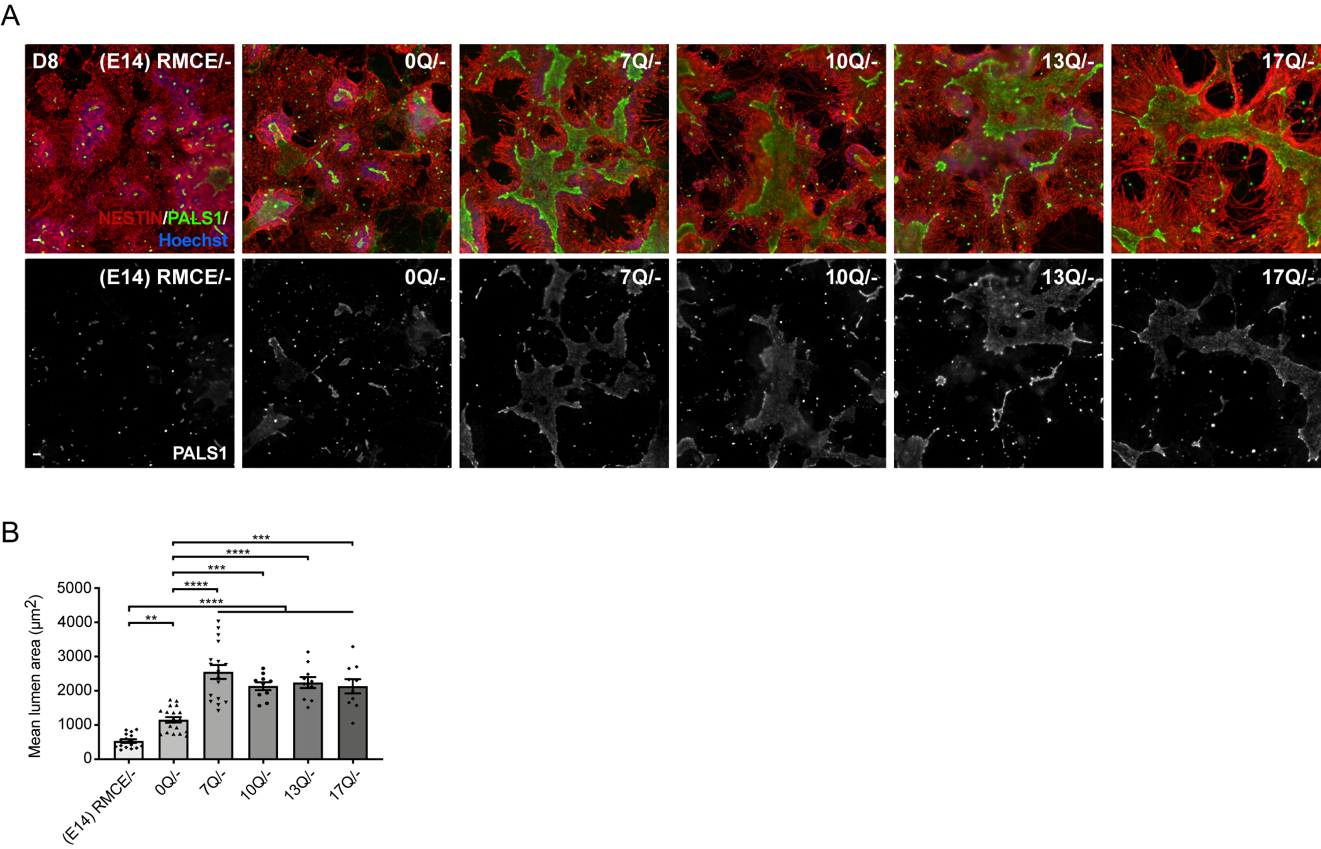


Figure S9: Effects of HTT polyQ tract deletion or expansion above 7Q in a (E14) ES cell-derived neural rosette formation assay*.*

A Representative images of rosette/lumen phenotype in E14 RMCE/-, 0Q/-, 7Q/-, 10Q/-, 13Q/-, 17Q/- cell lines stained for PALS1 and NESTIN at day 8 after neural induction. B Mean lumen area (μm^2^) of E14 RMCE/-, 0Q/-, 7Q/-, 10Q/-, 13Q/-, 17Q/- neural progenitor cultures automatically quantified with CellProfiler. Data are expressed as mean ± SEM from n≥5 independent neural differentiation experiments. Each dot represents the mean lumen area per well testing a pool of 2 clones for each edited cell line (see Supplementary dataset S25 for raw data and for the exact number of biological replicates per each genotype). ** P<0.01, *** P<0.001, **** P<0.0001, one-way ANOVA test followed by Tukey. All images were automatically acquired by IN Cell Analyzer 6000. The scale bars correspond to 50 μm.

**
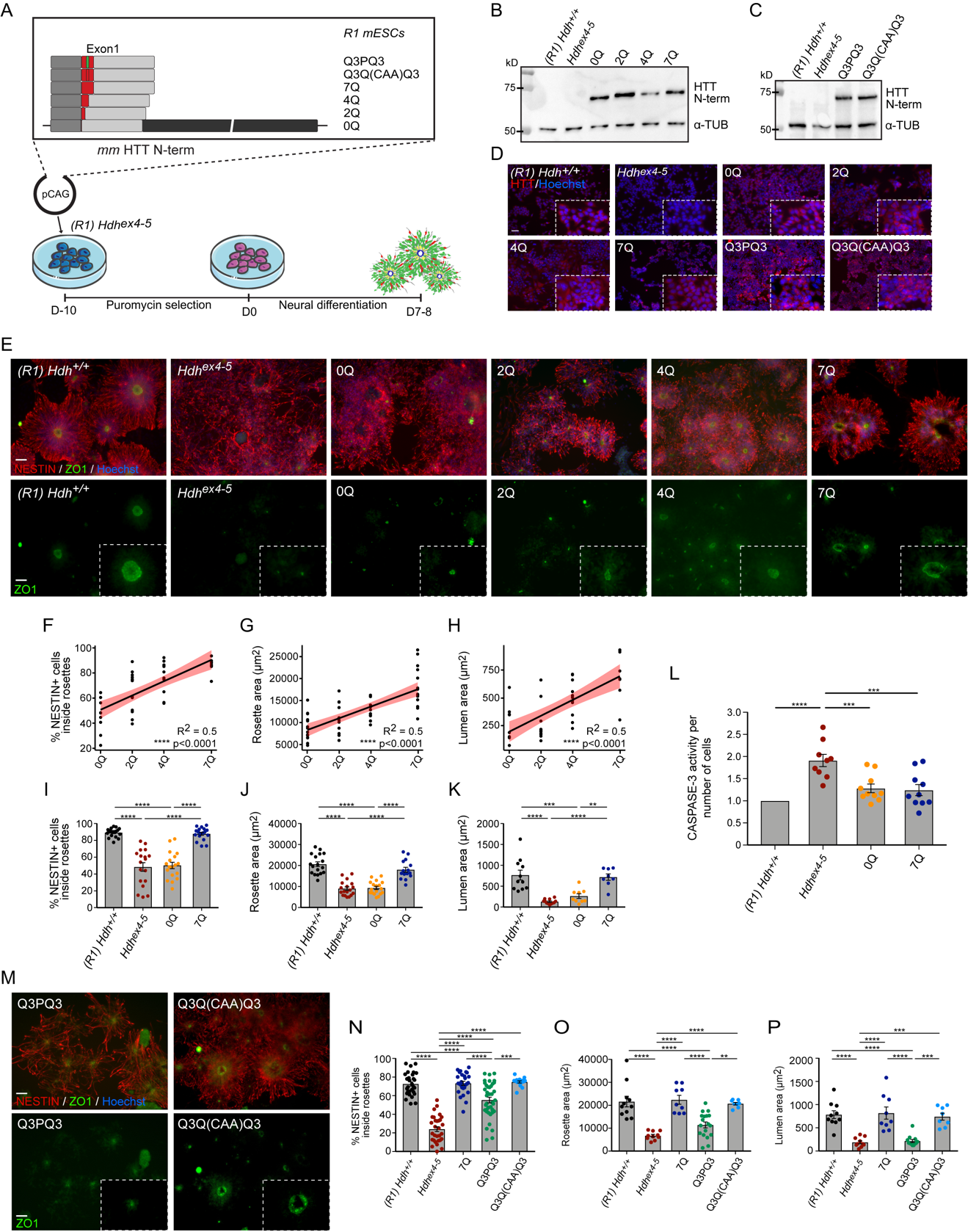
**

**Figure S10: Effects of HTT polyQ tract modulation in neural rosette formation assay with (R1) mES cell lines.**

**A** Scheme of the different polyQ tract modifications in the *Mus musculus* (*mm*) HTT N-terminus tested by rosette assay. **B-D** MAB-2166 (anti-HTT) Western blot (B and C) and immunostaining (D) in self-renewal conditions of R1 *Hdh^ex4-5^* cell lines that overexpress murine HTT N-termini with different polyQ lengths/substitutions. See **Supplementary dataset S24** for raw gel images. **E** Representative images of rosette/lumen phenotype in *Hdh^+/+^, Hdh^ex4-5^,* 0Q, 2Q, 4Q and 7Q neural progenitor cultures stained for ZO1 and NESTIN at day 7 after neural induction. **F-H** Linear regression lines and confidence intervals between Q length and rosette/lumen phenotypic parameters in 0Q, 2Q, 4Q and 7Q neural progenitor cultures. **I-K** Percentage of NESTIN+ cells inside the rosettes, rosette and lumen mean area in *Hdh^+/+^,* *Hdh^ex4-5^,* 0Q, and 7Q neural progenitor cultures. **L** CASPASE-3 activity in proliferating R1 *Hdh^+/+^, Hdh^ex4-5^,* 0Q and 7Q ES cells exposed to serum deprivation condition. Data are the mean ± SEM of ten independent experiments. **M** Representative images of rosette/lumen phenotype in Q3PQ3 and Q3Q(CAA)Q3 neural progenitor cultures. **N-P** Percentage of NESTIN+ cells within rosettes, rosette and lumen mean area in *Hdh^+/+^,* *Hdh^ex4-5^,* 7Q, Q3PQ3 and Q3Q(CAA)Q3 neural progenitor cultures. Data (in F to K and N to P) are expressed as mean ± SEM from n≥4 independent neural differentiation experiments. Each dot represents the mean values per well (see **Supplementary dataset S25** for raw data and for the exact number of biological replicates). ** P<0.01, *** P<0.001, **** P<0.0001, one-way ANOVA test followed by Bonferroni. The scale bars correspond to 50 μm. Insets are shown at 2X magnification.

**
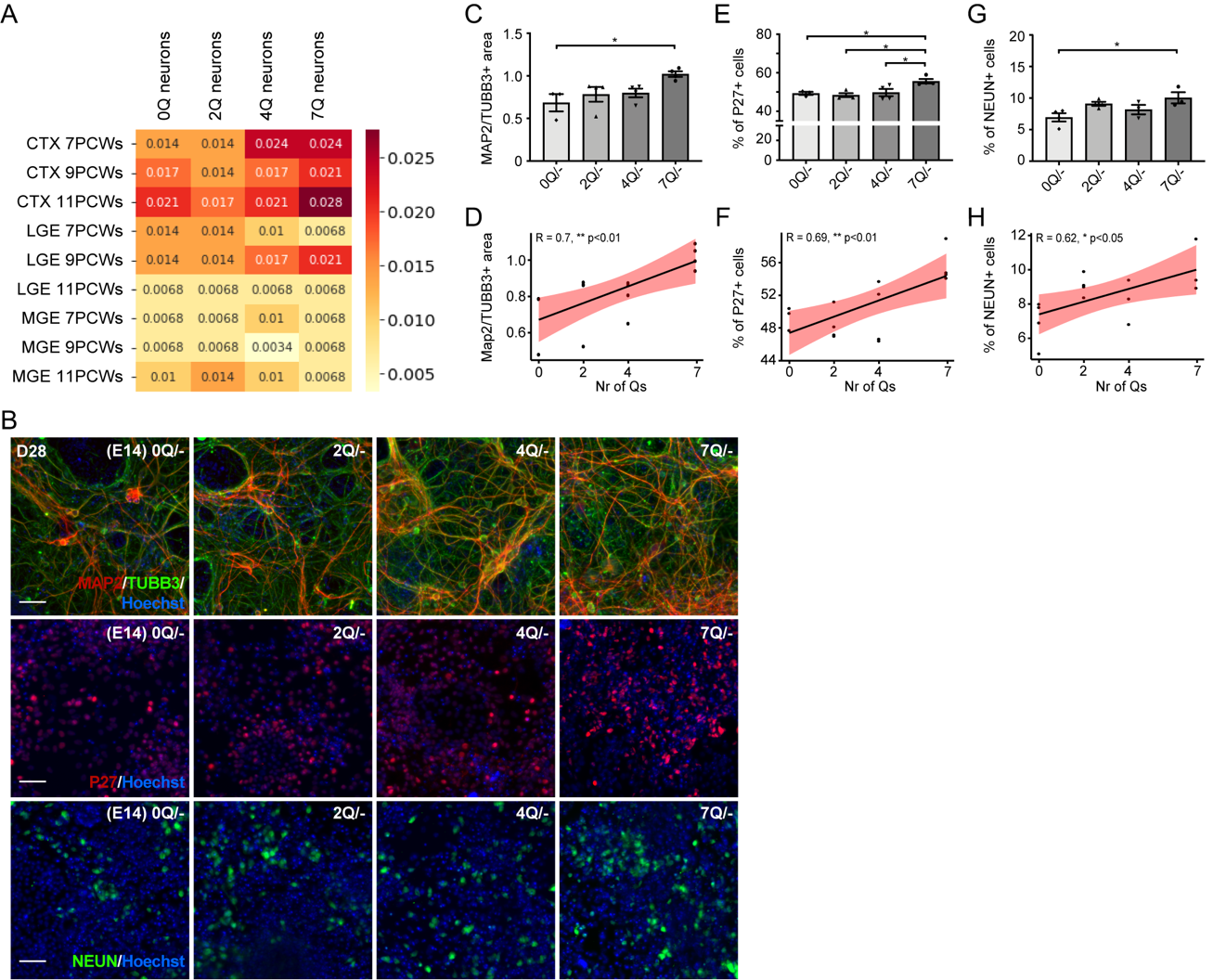
**

**Figure S11: Effects of non-pathogenic polyQ length modulation of HTT in maturing neurons.**

**A** Heatmap showing the “*Jaccard similarity*” index computed comparing the top 100 Differentially Expressed Genes (DEGs) between rosettes (early stage) and neurons (terminal stage) of 0Q, 2Q, 4Q and 7Q neuronal cultures with the top 100 unique DEGs of human cortical (CTX), striatal (Lateral Ganglionic Eminence, LGE) and pallidal (Medial Ganglionic Eminence, MGE) tissues. Colour gradient indicates “*Jaccard similarity*” index values (*red*: high similarity, *yellow*: low similarity); PCWs: post-conceptional weeks. **B** Representative images of neurons in (E14) 0Q/-, 2Q/-, 4Q/- and 7Q/- cultures stained for MAP2, TUBB3, P27 and NEUN at day 28 of neural differentiation. Hoechst dye was used to stain nuclei. **C** Quantification of MAP2^+^/TUBB3^+^ area in 0Q/-, 2Q/-, 4Q/- and 7Q/- neuronal cultures at day 28. **D** Linear regression line and confidence interval between Q length and MAP2^+^/TUBB3^+^ area in 0Q/-, 2Q/-, 4Q/- and 7Q/- neuronal cultures at day 28; Pearson correlation coefficients (R) and p-value are reported on the plot. **E** Counts of P27^+^ cells in 0Q/-, 2Q/-, 4Q/- and 7Q/- neuronal cultures at day 28. **F** Linear regression line and confidence interval between Q length and P27^+^ cells in 0Q/-, 2Q/-, 4Q/- and 7Q/- neuronal cultures at day 28; Pearson correlation coefficients (R) and p-value are reported on the plot. **G** Counts of NEUN^+^ cells in 0Q/-, 2Q/-, 4Q/- and 7Q/- neuronal cultures at day 28. **H** Linear regression line and confidence interval between Q length and NEUN^+^ cells in 0Q/-, 2Q/-, 4Q/- and 7Q/- neuronal cultures at day 28; Pearson correlation coefficients (R) and p-value are reported on the plot. All images of the neuronal analysis were automatically acquired by IN Cell Analyzer 6000 and automatically quantified with CellProfiler. Data in the bar plots (C), (E) and (G) are expressed as mean ± SEM from n≥3 independent neural terminal differentiation experiments. Each dot represents the mean value per well testing a pool of 2 clones for each edited cell line (see **Supplementary dataset S26** for raw data and for the exact number of biological replicates per each genotype). * P<0.05, ** P<0.01, *** P<0.001, **** P<0.0001, one-way ANOVA test followed by Tukey. The scale bars correspond to 50 μm.

**
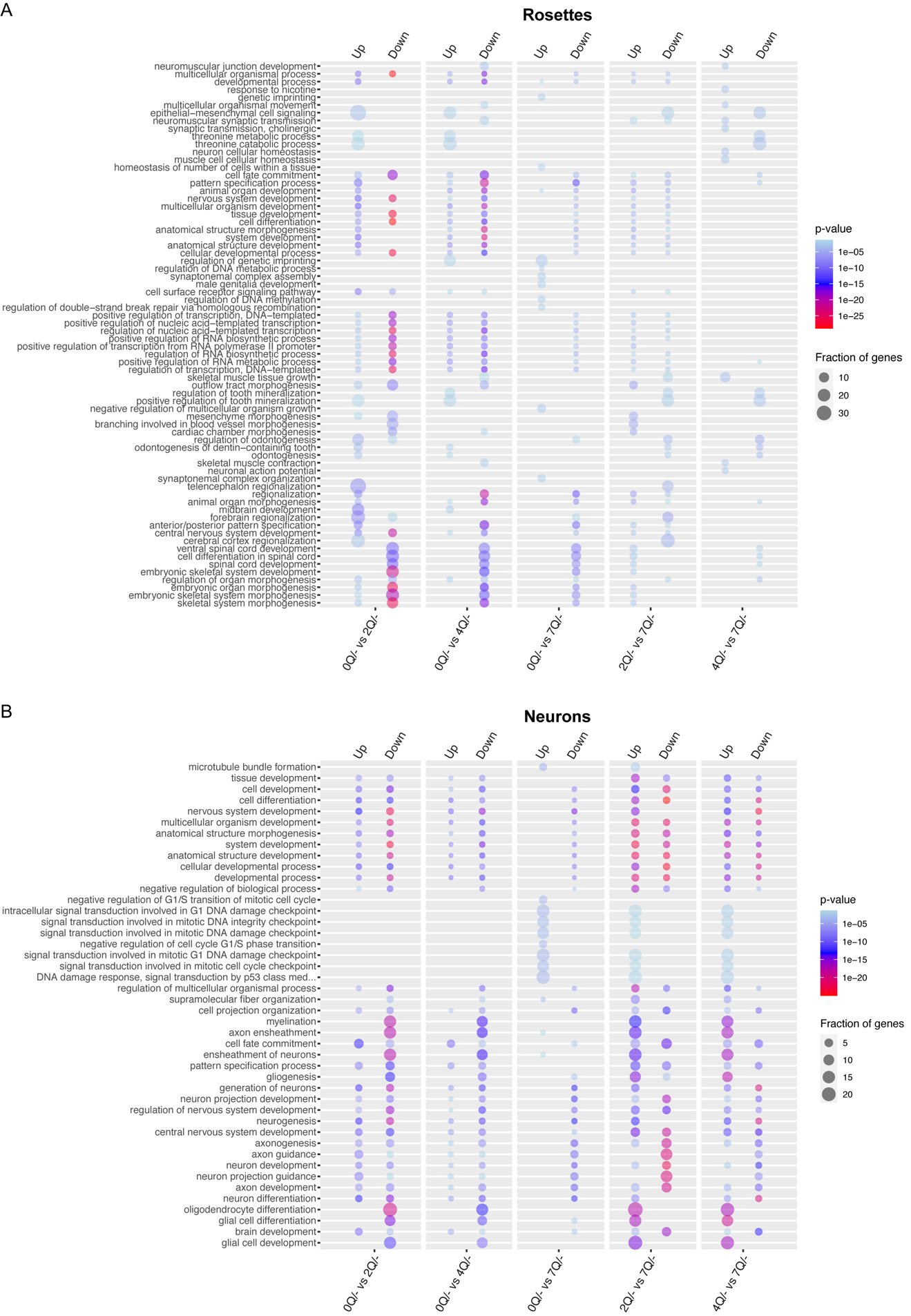
**

**Figure S12:** **Gene ontology enrichment analyses of all the pairwise comparisons among 0, 2, 4, and 7Q (E14) cells at the rosette and neuron stage.**

**A-B** GO dot plots of the top 10 significant GO terms (ranked on their p-value from a classic Fisher test) of the genes differentially expressed from each comparison at the stage of rosettes (A) or neurons (B). The plots display for each GO term the fraction of genes (size of the dot) and p-value (colour of the dot). Upregulated (up) and downregulated (down) DEGs were processed separately. For the full lists of DEGs see **Supplementary dataset S21**.

**LEGENDS FOR DATASETS S1 TO S26**

**Dataset S1 (separate file).** List of all 209 taxa considered in this study, source of sequences, accession numbers and selection criteria for the 158 subsequently analysed sequences.

**Dataset S2 (separate file).** Multi-fasta file for the 209 HTT sequences from taxa in data S1 used to generate the multi-alignment of Figure S1.

**Dataset S3 (separate file).** Nexus file with the MSA of HTT exon1 sequences from 163 taxa (74 mammals, 84 fishes, reptiles and birds, 5 ancestral species) used to count synonymous and non-synonymous substitutions.

**Dataset S4 (separate file).** Nexus file with the MSA of HTT exon1 sequences from 84 taxa with PolyQ stretch = 4Q, and their evolutionary tree in Newick format, used as input in FUBAR, SLAC and FEL analyses.

**Dataset S5 (separate file).** Nexus file with the MSA of HTT exon1 sequences from 74 taxa with PolyQ stretch > 4Q, and their evolutionary tree in Newick format, used as input in FUBAR, SLAC and FEL analyses.

**Dataset S6 (separate file).** List of the 53 taxa for which it was possible to retrieve both ATXN3 and HTT sequences. The file contains ATXN3 accession numbers.

**Dataset S7 (separate file).** Nexus file with the MSA of HTT exon1 sequences from 53 taxa from data S6 for which an ATXN3 sequence was also available. Evolutionary tree in Newick format. This file was used as input in FUBAR analysis of Figure S4.

**Dataset S8 (separate file).** Nexus file with the MSA of ATXN3 sequences from 53 taxa from data S6 for which an HTT sequence was also available. Evolutionary tree in Newick format. This file was used as input in FUBAR analysis of Figure S4.

**Dataset S9-S17 (separate files).** Nexus files with the MSA of AR, ATN1, ATXN1, ATXN2, ATXN7, CACNA1A, TBP, POU6F2, and ZNF384 sequences, respectively.

**Dataset S18 (separate file).** Full lists of polyQ-containing proteins with atypical LQ, LNI and PQ z-score values.

**Dataset S19 (separate file).** Fasta file with the *pHTT* sequence of *Callithrix jacchus*.

**Dataset S20 (separate file).** Raw data of *Callithrix jacchus pHTT* and *HTT* gene comparison.

**Dataset S21 (separate file).** Full lists of DEGs obtained from RNA-seq analyses.

**Dataset S22 (separate file).** Primers list and amplification protocols of *HTT* exon1 orthologs.

**Dataset S23 (separate file).** Genome editing primers/gRNAs list and RMCE sequence.

**Dataset S24 (separate file).** The uncropped PCR and western blot images.

**Dataset S25 (separate file).** Raw data of cell differentiation experiments (rosette stage).

**Dataset S26 (separate file).** Raw data of cell differentiation experiments (neuron stage).
