# Supplementary material for: The evolutionary history of the polyQ tract in huntingtin sheds light on its functional pro-neural activities
Source: Cell Death Differ. 2022 Jan 1;29(2):293–305. doi: 10.1038/s41418-021-00914-9 (PMC8817008; doi:10.1038/s41418-021-00914-9)
Supplement: Supplementary file 7 — PRE Authorship+form_spring nature - pre-acc journals [file 41418_2021_914_MOESM7_ESM.pdf]

SPRINGER NATURE

Change of authorship request form - Journals (pre-acceptance)

Important information. Please read.

- This form should be used by authors to request any change in authorship (adding/deleting authors) including changes in corresponding authors. This form should not be used for name changes. Please fully complete all sections. Use black ink and block capitals and provide each author's full name with the given name first followed by the family name.
- By signing this declaration, all authors guarantee that the order of the authors are in accordance with their scientific contribution, if applicable as different conventions apply per discipline, and that only authors have been added who made a meaningful contribution to the work.
- Please note, in author collaborations where there is formal agreement for representing the collaboration, it is sufficient for the representative or legal guarantor (usually the corresponding author) to complete and sign the Authorship Change Form on behalf of all authors, **next to the added/removed author(s). (Complete Section 3, followed by Section 6.)**
  In author collaborations where there is no formal agreement for representing the collaboration and **there are more than 10 authors**, one may sign for all, provided the signer appends correspondence that attests that each of the authors have agreed to the change **and the added/removed authors sign the form. (Complete Section 3, followed by Section 6.)**
- Please note, we cannot investigate or mediate any authorship disputes. If you are unable to obtain agreement from all authors (including those who you wish to be removed) you must refer the matter to your institution(s) for investigation. Please inform us if you need to do this.
- If you are not able to return a fully completed form within **30 days** of the date that it was sent to the author requesting the change, we may have to withdraw your manuscript. We cannot publish manuscripts where authorship has not been agreed by all authors (including those who have been removed).
- Incomplete forms will be rejected.
- Please return/upload this form, fully completed, to the Journals Editorial Office. The Journal and/or Publisher will consider the information you have provided to decide whether to approve the proposed change in authorship. We may decide to contact your institution for more information or undertake a further investigation, if appropriate, before making a final decision.

SPRINGER NATURE Springer Nature is one of the world's leading global research, educational and professional publishers, created in May 2015 through the combination of Nature Publishing Group, Palgrave Macmillan, Macmillan Education and Springer Science+Business Media.

SPRINGER NATURE Change of authorship request form - Journals (pre-acceptance)

Section 1: Please provide the current title of manuscript

Manuscript ID no.: CDD-21-1356RR

Title: THE EVOLUTIONARY HISTORY OF THE POLYQ TRACT IN HUNTINGTIN SHEDS LIGHT ON ITS FUNCTIONAL PRO-NEURAL ACTIVITIES

Section 2: Please provide the previous authorship, in the order shown on the manuscript before the changes were introduced. Please indicate the corresponding author by adding (CA) behind the name.

	First name(s)	Family name	ORCID or SCOPUS id, if available
1st author	RAFFAELE	IENNACO	
2nd author	GIULIO	FORMENTI	
3rd author	CAMILLA	TROVESI	
4th author	RICCARDO LORENZO	ROSSI	
5th author	CHIARA	ZUCCATO	
6th author	TIZIANA	LISCHETTI	
7th author	VITTORIA DICKINSON	BOCCHI	
8th author	ANDREA	SCOLZ	
9th author	CRISTINA	MARTINEZ-LABARGA	
10th author	OLGA	RICKARDS	

Please use an additional sheet if there are more than 10 authors.

SPRINGER NATURE Springer Nature is one of the world's leading global research, educational and professional publishers, created in May 2015 through the combination of Nature Publishing Group, Palgrave Macmillan, Macmillan Education and Springer Science+Business Media.

SPRINGER NATURE

Change of authorship request form - Journals (pre-acceptance)

Section 1: Please provide the current title of manuscript

Manuscript ID no.: CDD-21-1356RR

Title: THE EVOLUTIONARY HISTORY OF THE POLYQ TRACT IN HUNTINGTIN SHEDS LIGHT ON ITS FUNCTIONAL PRO-NEURAL ACTIVITIES

Section 2: Please provide the previous authorship, in the order shown on the manuscript before the changes were introduced. Please indicate the corresponding author by adding (CA) behind the name.

		First name(s)	Family name	ORCID or SCOPUS id, if available
11	1st author	MICHELA	PACIFICO	
12	2nd author	ANGELICA	CROTTINI	
13	3rd author	ANDERS PAPE	MØLLER	
14	4th author	GIULIO	PAVESI	
15	5th author	DAVID STEPHEN	HORNER	
16	6th author	NICOLA	SAINO	
17	7th author	ELENA (CA)	CATTANEO	0000-0002-0755-4917
	~~8th author~~			
	~~9th author~~			
	~~10th author~~			

Please use an additional sheet if there are more than 10 authors.

SPRINGER NATURE Springer Nature is one of the world's leading global research, educational and professional publishers, created in May 2015 through the combination of Nature Publishing Group, Palgrave Macmillan, Macmillan Education and Springer Science+Business Media.

SPRINGER NATURE

Change of authorship request form - Journals (pre-acceptance)

Section 3: Please provide a justification for change. Please use this section to explain your reasons for changing the authorship of your manuscript, e.g. what necessitated the change in authorship? Please refer to the (journal) policy pages for more information about authorship. Please explain why omitted authors were not originally included and/or why authors were removed on the submitted manuscript.

The addition of two other authors is due to the fact that they were involved in the original planning and design of the master RMCE line which should have been described in a separate paper dealing with the effect of an expanded (mutant) CAG repeat in HTT on cell physiology. Since - as of today - this other manuscript has not yet been submitted, the acceptance of the current paper by Iennaco et al in CDD becomes the first report of the generation of the RMCE lines. For this reason it is important to include here both Dr. Thomas Vogt and Dr. Richard Chen as they have contributed to the original discussion and design of the strategy that led to the production of the RMCE master line which - in the paper by Iennaco et al - was further developed to carry CAG repeats in the lower normal range.

Section 4: Proposed new authorship. Please provide your new authorship list in the order you would like it to appear on the manuscript. Please indicate the corresponding author by adding (CA) behind the name. If the Corresponding Author has changed, please indicate the reason under section 3.

	First name(s)	Family name (this name will appear in full on the final publication and will be searchable in various abstract and indexing databases)	Affiliated institute	E-mail address
1st author	RAFFAELE	IENNACO	UNIVERSITY OF MILAN	raffaele.iennaco@unimi.it
2nd author	GIULIO	FORMENTI	UNIVERSITY OF MILAN	giulio.formenti@guest.unimi.it
3rd author	CAMILLA	TROVESI	UNIVERSITY OF MILAN	camilla.trovesi@unimi.it
4th author	RICCARDO LORENZO	ROSSI	INGM	rossi@ingm.org
5th author	CHIARA	ZUCCATO	UNIVERSITY OF MILAN	chiara.zuccato@unimi.it
6th author	TIZIANA	LISCHETTI	UNIVERSITY OF MILAN	tiziana.lischetti@unimi.it
7th author	VITTORIA DICKINSON	BOCCHI	UNIVERSITY OF MILAN	vittoria.bocchi@unimi.it
8th author	ANDREA	SCOLZ	UNIVERSITY OF MILAN	andrea.scolz@unimi.it
9th author	CRISTINA	MARTINEZ-LABARGA	UNIVERSITY OF ROME TOR VERGATA	martine@uniroma2.it
10th author	OLGA	RICKARDS	UNIVERSITY OF ROME TOR VERGATA	rickards@uniroma2.it

Please use an additional sheet if there are more than 10 authors.

SPRINGER NATURE Springer Nature is one of the world's leading global research, educational and professional publishers, created in May 2015 through the combination of Nature Publishing Group, Palgrave Macmillan, Macmillan Education and Springer Science+Business Media.

SPRINGER NATURE

Change of authorship request form - Journals (pre-acceptance)

Section 3: Please provide a justification for change. Please use this section to explain your reasons for changing the authorship of your manuscript, e.g. what necessitated the change in authorship? Please refer to the (journal) policy pages for more information about authorship. Please explain why omitted authors were not originally included and/or why authors were removed on the submitted manuscript.

The addition of two other authors is due to the fact that they were involved in the original planning and design of the master RMCE line which should have been described in a separate paper dealing with the effect of an expanded (mutant) CAG repeat in HTT on cell physiology. Since - as of today - this other manuscript has not yet been submitted, the acceptance of the current paper by lennaco et al in CDD becomes the first report of the generation of the RMCE lines. For this reason it is important to include here both Dr. Thomas Vogt and Dr. Richard Chen as they have contributed to the original discussion and design of the strategy that led to the production of the RMCE master line which - in the paper by lennaco et al - was further developed to carry CAG repeats in the lower normal range.

Section 4: Proposed new authorship. Please provide your new authorship list in the order you would like it to appear on the manuscript. Please indicate the corresponding author by adding (CA) behind the name. <u>If the Corresponding Author has changed, please indicate the reason under section 3.</u>

		First name(s)	Family name (this name will appear in full on the final publication and will be searchable in various abstract and indexing databases)	Affiliated institute	E-mail address
11	1st author	MICHELA	PACIFICO	UNIVERSITY OF MILAN	mhlpacifico@gmail.com
12	2nd author	ANGELICA	CROTTINI	UNIVERSIDADE DO PORTO	tiliquait@yahoo.it
13	3rd author	ANDERS PAPE	MØLLER	UNIVERSITE' PARIS-SACLAY	anders.moller@u-psud.fr
14	4th author	RICHARD Z.	CHEN	CHDI FOUNDATION	richard.chen@chdifoundation.org
15	5th author	THOMAS F.	VOGT	CHDI FOUNDATION	thomas.vogt@chdifoundation.org
16	6th author	GIULIO	PAVESI	UNIVERSITY OF MILAN	giulio.pavesi@unimi.it
17	7th author	DAVID STEPHEN	HORNER	UNIVERSITY OF MILAN	david.horner@unimi.it
18	8th author	NICOLA	SAINO	UNIVERSITY OF MILAN	nicola.saino@unimi.it
19	9th author	ELENA (CA)	CATTANEO	UNIVERSITY OF MILAN	elena.cattaneo@unimi.it
	~~10th author~~				

Please use an additional sheet if there are more than 10 authors.

SPRINGER NATURE Springer Nature is one of the world's leading global research, educational and professional publishers, created in May 2015 through the combination of Nature Publishing Group, Palgrave Macmillan, Macmillan Education and Springer Science+Business Media.

SPRINGER NATURE

Change of authorship request form - Journals (pre-acceptance)

Section 5: Author contribution, Acknowledgement and Disclosures. Please use this section to provide a new disclosure statement and, if appropriate, acknowledge any contributors who have been removed as authors and ensure you state what contribution any new authors made (if applicable per the journal or book (series) policy). **Please ensure these are updated in your manuscript - after approval of the change(s) - as our production department will not transfer the information in this form to your manuscript.**

New acknowledgements:

/

New Disclosures (financial and non-financial interests, funding):

/

New Author Contributions statement (if applicable per the journal policy):

R.Z.C. (Richard Z. Chen) contributed to the design of the experimental strategy to knock-out HTT in E14 mES cells T.F.V. (Thomas F. Vogt) conceived RMCE cell platform

State 'Not applicable' if there are no new authors.

SPRINGER NATURE Springer Nature is one of the world's leading global research, educational and professional publishers, created in May 2015 through the combination of Nature Publishing Group, Palgrave Macmillan, Macmillan Education and Springer Science+Business Media.

SPRINGER NATURE

Change of authorship request form - Journals (pre-acceptance)

Section 6: Declaration of agreement. *All* authors, unchanged, new and removed *must* sign this declaration.
(NB: Please print the form, (docu)-sign and return/upload a scanned copy. Please note that signatures that have been inserted as an image file are acceptable as long as it is handwritten. Typed names in the signature box are unacceptable.) *** Please delete as appropriate. Delete all of the bold if you were on the original authorship list and are remaining as an author.**

	First name	Family name		Signature	Date
1st author	RAFFAELE	IENNACO	I agree to the proposed new authorship shown in section 4 ~~/and the addition/removal*of my name to the authorship list /and the proposed change in corresponding author~~	Raffaele Iennaco	mm/dd/yyyy 11/10/2021
2nd author	GIULIO	FORMENTI	I agree to the proposed new authorship shown in section 4 ~~/and the addition/removal*of my name to the authorship list /and the proposed change in corresponding author~~		
3rd author	CAMILLA	TROVESI	I agree to the proposed new authorship shown in section 4 ~~/and the addition/removal*of my name to the authorship list /and the proposed change in corresponding author~~		
4th authors	RICCARDO LORENZO	ROSSI	I agree to the proposed new authorship shown in section 4 ~~/and the addition/removal*of my name to the authorship list /and the proposed change in corresponding author~~	Riccardo L. Rossi	mm/dd/yyyy 11/10/2021
5th author	CHIARA	ZUCCATO	I agree to the proposed new authorship shown in section 4 ~~/and the addition/removal*of my name to the authorship list /and the proposed change in corresponding author~~	Chiara Zuccato	mm/dd/yyyy 11/10/2021
6th author	TIZIANA	LISCHETTI	I agree to the proposed new authorship shown in section 4 ~~/and the addition/removal*of my name to the authorship list /and the proposed change in corresponding author~~		
7th author	VITTORIA DICKINSON	BOCCHI	I agree to the proposed new authorship shown in section 4 ~~/and the addition/removal*of my name to the authorship list /and the proposed change in corresponding author~~		

SPRINGER NATURE Springer Nature is one of the world's leading global research, educational and professional publishers, created in May 2015 through the combination of Nature Publishing Group, Palgrave Macmillan, Macmillan Education and Springer Science+Business Media.

SPRINGER NATURE Change of authorship request form - Journals (pre-acceptance)

Section 6: Declaration of agreement. ***All*** **authors, unchanged, new and removed** ***must*** **sign this declaration.**
(NB: Please print the form, (docu)-sign and return/upload a scanned copy. Please note that signatures that have been inserted as an image file are acceptable as long as it is handwritten. Typed names in the signature box are unacceptable.) *** Please delete as appropriate. Delete all of the bold if you were on the original authorship list and are remaining as an author.**

	First name	Family name		Signature	Date
1st author	RAFFAELE	IENNACO	I agree to the proposed new authorship shown in section 4 ~~/and the addition/removal*of my name to the authorship list /and the proposed change in corresponding author~~	Raffaele Iennaco	mm/dd/yyyy 11/10/202[illegible]
2nd author	GIULIO	FORMENTI	I agree to the proposed new authorship shown in section 4 ~~/and the addition/removal*of my name to the authorship list /and the proposed change in corresponding author~~	Giulio Formenti	dd/mm/yyyy 22/11/2021
3rd author	CAMILLA	TROVESI	I agree to the proposed new authorship shown in section 4 ~~/and the addition/removal*of my name to the authorship list /and the proposed change in corresponding author~~		
4th authors	RICCARDO LORENZO	ROSSI	I agree to the proposed new authorship shown in section 4 ~~/and the addition/removal*of my name to the authorship list /and the proposed change in corresponding author~~	Riccardo L. Rossi	mm/dd/yyyy 11/10/2021
5th author	CHIARA	ZUCCATO	I agree to the proposed new authorship shown in section 4 ~~/and the addition/removal*of my name to the authorship list /and the proposed change in corresponding author~~	Chiara Zuccato	mm/dd/yyyy 11/10/2021
6th author	TIZIANA	LISCHETTI	I agree to the proposed new authorship shown in section 4 ~~/and the addition/removal*of my name to the authorship list /and the proposed change in corresponding author~~		
7th author	VITTORIA DICKINSON	BOCCHI	I agree to the proposed new authorship shown in section 4 ~~/and the addition/removal*of my name to the authorship list /and the proposed change in corresponding author~~		

SPRINGER NATURE Springer Nature is one of the world's leading global research, educational and professional publishers, created in May 2015 through the combination of Nature Publishing Group, Palgrave Macmillan, Macmillan Education and Springer Science+Business Media.

SPRINGER NATURE

Change of authorship request form - Journals (pre-acceptance)

Section 6: Declaration of agreement. *All* authors, unchanged, new and removed *must* sign this declaration.
(NB: Please print the form, (docu)-sign and return/upload a scanned copy. Please note that signatures that have been inserted as an image file are acceptable as long as it is handwritten. Typed names in the signature box are unacceptable.) * **Please delete as appropriate. Delete all of the bold if you were on the original authorship list and are remaining as an author.**

	First name	Family name		Signature	Date
1st author	RAFFAELE	IENNACO	I agree to the proposed new authorship shown in section 4 ~~/and the addition/removal*of my name to the authorship list /and the proposed change in corresponding author~~	Raffaele Iennaco	mm/dd/yyyy 11/10/2021
2nd author	GIULIO	FORMENTI	I agree to the proposed new authorship shown in section 4 /~~and the addition/removal*of my name to the authorship list /and the proposed change in corresponding author~~		
3rd author	CAMILLA	TROVESI	I agree to the proposed new authorship shown in section 4 /~~and the addition/removal*of my name to the authorship list /and the proposed change in corresponding author~~	Camilla Trovesi	mm/dd/yyyy 11/11/2021
4th authors	RICCARDO LORENZO	ROSSI	I agree to the proposed new authorship shown in section 4 /~~and the addition/removal*of my name to the authorship list /and the proposed change in corresponding author~~	Riccardo L. Rossi	mm/dd/yyyy 11/10/2021
5th author	CHIARA	ZUCCATO	I agree to the proposed new authorship shown in section 4 /~~and the addition/removal*of my name to the authorship list /and the proposed change in corresponding author~~	Chiara Zuccato	mm/dd/yyyy 11/10/2021
6th author	TIZIANA	LISCHETTI	I agree to the proposed new authorship shown in section 4 /~~and the addition/removal*of my name to the authorship list /and the proposed change in corresponding author~~		
7th author	VITTORIA DICKINSON	BOCCHI	I agree to the proposed new authorship shown in section 4 /~~and the addition/removal*of my name to the authorship list /and the proposed change in corresponding author.~~		

SPRINGER NATURE Springer Nature is one of the world's leading global research, educational and professional publishers, created in May 2015 through the combination of Nature Publishing Group, Palgrave Macmillan, Macmillan Education and Springer Science+Business Media.

SPRINGER NATURE

Change of authorship request form - Journals (pre-acceptance)

Section 6: Declaration of agreement. *All* authors, unchanged, new and removed *must* sign this declaration.
(NB: Please print the form, (docu)-sign and return/upload a scanned copy. Please note that signatures that have been inserted as an image file are acceptable as long as it is handwritten. Typed names in the signature box are unacceptable.)* **Please delete as appropriate. Delete all of the bold if you were on the original authorship list and are remaining as an author.**

	First name	Family name		Signature	Date
1st author	RAFFAELE	IENNACO	I agree to the proposed new authorship shown in section 4 ~~/and the addition/removal* of my name to the authorship list /and the proposed change in corresponding author~~	Raffaele Iennaco	mm/dd/yyyy 11/10/2021
2nd author	GIULIO	FORMENTI	I agree to the proposed new authorship shown in section 4 ~~/and the addition/removal* of my name to the authorship list /and the proposed change in corresponding author~~		
3rd author	CAMILLA	TROVESI	I agree to the proposed new authorship shown in section 4 ~~/and the addition/removal* of my name to the authorship list /and the proposed change in corresponding author~~		
4th authors	RICCARDO LORENZO	ROSSI	I agree to the proposed new authorship shown in section 4 ~~/and the addition/removal* of my name to the authorship list /and the proposed change in corresponding author~~	[illegible]	mm/dd/yyyy 11/10/2021
5th author	CHIARA	ZUCCATO	I agree to the proposed new authorship shown in section 4 ~~/and the addition/removal* of my name to the authorship list /and the proposed change in corresponding author~~	Chiara Zuccato	mm/dd/yyyy 11/10/2021
6th author	TIZIANA	LISCHETTI	I agree to the proposed new authorship shown in section 4 ~~/and the addition/removal* of my name to the authorship list /and the proposed change in corresponding author~~	[illegible]	mm/dd/yyyy 11/11/2021
7th author	VITTORIA DICKINSON	BOCCHI	I agree to the proposed new authorship shown in section 4 ~~/and the addition/removal* of my name to the authorship list /and the proposed change in corresponding author~~		

SPRINGER NATURE Springer Nature is one of the world's leading global research, educational and professional publishers, created in May 2015 through the combination of Nature Publishing Group, Palgrave Macmillan, Macmillan Education and Springer Science+Business Media.

SPRINGER NATURE

Change of authorship request form - Journals (pre-acceptance)

Section 6: Declaration of agreement. *All* authors, unchanged, new and removed *must* sign this declaration.
(NB: Please print the form, (docu)-sign and return/upload a scanned copy. Please note that signatures that have been inserted as an image file are acceptable as long as it is handwritten. Typed names in the signature box are unacceptable.) * Please delete as appropriate. Delete all of the bold if you were on the original authorship list and are remaining as an author.

	First name	Family name		Signature	Date
1st author	RAFFAELE	IENNACO	I agree to the proposed new authorship shown in section 4 / ~~**and the addition/removal* of my name to the authorship list / and the proposed change in corresponding author**~~	Raffaele Iennaco	mm/dd/yyyy 11/10/2021
2nd author	GIULIO	FORMENTI	I agree to the proposed new authorship shown in section 4 / ~~**and the addition/removal* of my name to the authorship list / and the proposed change in corresponding author**~~		
3rd author	CAMILLA	TROVESI	I agree to the proposed new authorship shown in section 4 / ~~**and the addition/removal* of my name to the authorship list / and the proposed change in corresponding author**~~		
4th authors	RICCARDO LORENZO	ROSSI	I agree to the proposed new authorship shown in section 4 / ~~**and the addition/removal* of my name to the authorship list / and the proposed change in corresponding author**~~	[illegible]	mm/dd/yyyy 11/10/2021
5th author	CHIARA	ZUCCATO	I agree to the proposed new authorship shown in section 4 / ~~**and the addition/removal* of my name to the authorship list / and the proposed change in corresponding author**~~	Chiara Zuccato	mm/dd/yyyy 11/10/2021
6th author	TIZIANA	LISCHETTI	I agree to the proposed new authorship shown in section 4 / ~~**and the addition/removal* of my name to the authorship list / and the proposed change in corresponding author**~~		
7th author	VITTORIA DICKINSON	BOCCHI	I agree to the proposed new authorship shown in section 4 / ~~**and the addition/removal* of my name to the authorship list / and the proposed change in corresponding author**~~	[illegible]	mm/dd/yyyy 11/11/2021

SPRINGER NATURE Springer Nature is one of the world's leading global research, educational and professional publishers, created in May 2015 through the combination of Nature Publishing Group, Palgrave Macmillan, Macmillan Education and Springer Science+Business Media.

SPRINGER NATURE

Change of authorship request form - Journals (pre-acceptance)

	First name	Family name		Signature	Date mm/dd/yyyy
8th author	ANDREA	SCOLZ	I agree to the proposed new authorship shown in section 4 /~~and the addition/removal*of my name to the authorship list /and the proposed change in corresponding author~~		11/10/2021
9th author	CRISTINA	MARTINEZ - LABARGA	I agree to the proposed new authorship shown in section 4 /~~and the addition/removal*of my name to the authorship list /and the proposed change in corresponding author~~		
10th author	OLGA	RICKARDS	I agree to the proposed new authorship shown in section 4 /~~and the addition/removal*of my name to the authorship list /and the proposed change in corresponding author~~		

Please use an additional sheet if there are more than 10 authors.

In case of author collaborations with formal agreement:

	Name of consortium/consortia	First name	Family name		Signature	Date
Representative/legal guarantor				I agree to the proposed new authorship shown in section 4 **/and the addition/removal*of my name to the authorship list** /and the proposed change in corresponding author		

Both added/removed authors should complete the information in the first table under Section 6.

---- End of form ----

SPRINGER NATURE Springer Nature is one of the world's leading global research, educational and professional publishers, created in May 2015 through the combination of Nature Publishing Group, Palgrave Macmillan, Macmillan Education and Springer Science+Business Media.

SPRINGER NATURE

Change of authorship request form - Journals (pre-acceptance)

	First name	Family name		Signature	Date
8th author	ANDREA	SCOLZ	I agree to the proposed new authorship shown in section 4 /~~and the addition/removal*of my name to the authorship list /and the proposed change in corresponding author~~	[illegible]	mm/dd/yyyy 11/10/2021
9th author	CRISTINA	MARTINEZ - LABARGA	I agree to the proposed new authorship shown in section 4 /~~and the addition/removal*of my name to the authorship list /and the proposed change in corresponding author~~	[illegible]	mm/dd/yyyy 11/11/2021
10th author	OLGA	RICKARDS	I agree to the proposed new authorship shown in section 4 /~~and the addition/removal*of my name to the authorship list /and the proposed change in corresponding author~~	[illegible]	mm/dd/yyyy 11/11/2021

Please use an additional sheet if there are more than 10 authors.

In case of author collaborations with formal agreement:

	Name of consortium/consortia	First name	Family name		Signature	Date
Representative/legal guarantor				I agree to the proposed new authorship shown in section 4 /and the addition/removal*of my name to the authorship list /and the proposed change in corresponding author		

Both added/removed authors should complete the information in the first table under Section 6.

---- End of form ----

SPRINGER NATURE Springer Nature is one of the world's leading global research, educational and professional publishers, created in May 2015 through the combination of Nature Publishing Group, Palgrave Macmillan, Macmillan Education and Springer Science+Business Media.

Change of authorship request form - Journals (pre-acceptance)

Section 6: Declaration of agreement. *All* authors, unchanged, new and removed *must* sign this declaration.
(NB: Please print the form, (docu)-sign and return/upload a scanned copy. Please note that signatures that have been inserted as an image file are acceptable as long as it is handwritten. Typed names in the signature box are unacceptable.) * Please delete as appropriate. Delete all of the bold if you were on the original authorship list and are remaining as an author.

	First name	Family name		Signature	Date dd/mm/yyyy
~~1st~~ author 11	MICHELA	PACIFICO	I agree to the proposed new authorship shown in section 4 /~~and the addition/removal* of my name to the authorship list /and the proposed change in corresponding author~~	Michela Pacifico	16/11/2021
~~2nd~~ author 12	ANGELICA	CROTTINI	I agree to the proposed new authorship shown in section 4 /~~and the addition/removal* of my name to the authorship list /and the proposed change in corresponding author~~		
~~3rd~~ author 13	ANDERS PAPE	MØLLER	I agree to the proposed new authorship shown in section 4 /~~and the addition/removal* of my name to the authorship list /and the proposed change in corresponding author~~		
~~4th~~ authors 14	RICHARD Z.	CHEN	I agree to the proposed new authorship shown in section 4 /**and the addition/~~removal~~* of my name to the authorship list** /~~and the proposed change in corresponding author~~		
~~5th~~ author 15	THOMAS F.	VOGT	I agree to the proposed new authorship shown in section 4 /**and the addition/~~removal~~* of my name to the authorship list** /~~and the proposed change in corresponding author~~		
~~6th~~ author 16	GIULIO	PAVESI	I agree to the proposed new authorship shown in section 4 /~~and the addition/removal* of my name to the authorship list /and the proposed change in corresponding author~~		
~~7th~~ author 17	DAVID STEPHEN	HORNER	I agree to the proposed new authorship shown in section 4 /~~and the addition/removal* of my name to the authorship list /and the proposed change in corresponding author~~		

SPRINGER NATURE Springer Nature is one of the world's leading global research, educational and professional publishers, created in May 2015 through the combination of Nature Publishing Group, Palgrave Macmillan, Macmillan Education and Springer Science+Business Media.

SPRINGER NATURE

Change of authorship request form - Journals (pre-acceptance)

Section 6: Declaration of agreement. *All* **authors, unchanged, new and removed** ***must*** **sign this declaration.**
(NB: Please print the form, (docu)-sign and return/upload a scanned copy. Please note that signatures that have been inserted as an image file are acceptable as long as it is handwritten. Typed names in the signature box are unacceptable.) *** Please delete as appropriate. Delete all of the bold if you were on the original authorship list and are remaining as an author.**

	First name	Family name		Signature	Date
~~1st~~ author 11	MICHELA	PACIFICO	I agree to the proposed new authorship shown in section 4 /~~and the addition/removal* of my name to the authorship list /and the proposed change in corresponding author~~		
~~2nd~~ author 12	ANGELICA	CROTTINI	I agree to the proposed new authorship shown in section 4 /~~and the addition/removal* of my name to the authorship list /and the proposed change in corresponding author~~	Angelica Crottini	mm/dd/yyyy 11/13/2021
~~3rd~~ author 13	ANDERS PAPE	MØLLER	I agree to the proposed new authorship shown in section 4 /~~and the addition/removal* of my name to the authorship list /and the proposed change in corresponding author~~		
~~4th~~ authors 14	RICHARD Z.	CHEN	I agree to the proposed new authorship shown in section 4 /**and the addition/~~removal~~* of my name to the authorship list** /~~and the proposed change in corresponding author~~		
~~5th~~ author 15	THOMAS F.	VOGT	I agree to the proposed new authorship shown in section 4 /**and the addition/~~removal~~* of my name to the authorship list** /~~and the proposed change in corresponding author~~		
~~6th~~ author 16	GIULIO	PAVESI	I agree to the proposed new authorship shown in section 4 /~~and the addition/removal* of my name to the authorship list /and the proposed change in corresponding author~~		
~~7th~~ author 17	DAVID STEPHEN	HORNER	I agree to the proposed new authorship shown in section 4 /~~and the addition/removal* of my name to the authorship list /and the proposed change in corresponding author~~		

SPRINGER NATURE Springer Nature is one of the world's leading global research, educational and professional publishers, created in May 2015 through the combination of Nature Publishing Group, Palgrave Macmillan, Macmillan Education and Springer Science+Business Media.

SPRINGER NATURE

Change of authorship request form - Journals (pre-acceptance)

Section 6: Declaration of agreement. *All* authors, unchanged, new and removed *must* sign this declaration.
(NB: Please print the form, (docu)-sign and return/upload a scanned copy. Please note that signatures that have been inserted as an image file are acceptable as long as it is handwritten. Typed names in the signature box are unacceptable.) * Please delete as appropriate. Delete all of the bold if you were on the original authorship list and are remaining as an author.

	First name	Family name		Signature	Date
~~1st~~ author 11	MICHELA	PACIFICO	I agree to the proposed new authorship shown in section 4 /~~and the addition/removal* of my name to the authorship list /and the proposed change in corresponding author~~		
~~2nd~~ author 12	ANGELICA	CROTTINI	I agree to the proposed new authorship shown in section 4 /~~and the addition/removal* of my name to the authorship list /and the proposed change in corresponding author~~		
~~3rd~~ author 13	ANDERS PAPE	MØLLER	I agree to the proposed new authorship shown in section 4 /~~and the addition/removal* of my name to the authorship list /and the proposed change in corresponding author~~	AP Møller	dd/mm/yyyy 12/1/2021
~~4th~~ authors 14	RICHARD Z.	CHEN	I agree to the proposed new authorship shown in section 4 /and the addition/~~removal*~~ of my name to the authorship list /~~and the proposed change in corresponding author~~		
~~5th~~ author 15	THOMAS F.	VOGT	I agree to the proposed new authorship shown in section 4 /and the addition/~~removal*~~ of my name to the authorship list /~~and the proposed change in corresponding author~~		
~~6th~~ author 16	GIULIO	PAVESI	I agree to the proposed new authorship shown in section 4 /~~and the addition/removal* of my name to the authorship list /and the proposed change in corresponding author~~		
~~7th~~ author 17	DAVID STEPHEN	HORNER	I agree to the proposed new authorship shown in section 4 /~~and the addition/removal* of my name to the authorship list /and the proposed change in corresponding author~~		

SPRINGER NATURE Springer Nature is one of the world's leading global research, educational and professional publishers, created in May 2015 through the combination of Nature Publishing Group, Palgrave Macmillan, Macmillan Education and Springer Science+Business Media.

SPRINGER NATURE Change of authorship request form - Journals (pre-acceptance)

Section 6: Declaration of agreement. ***All*** **authors, unchanged, new and removed** ***must*** **sign this declaration.**
(NB: Please print the form, (docu)-sign and return/upload a scanned copy. Please note that signatures that have been inserted as an image file are acceptable as long as it is handwritten. Typed names in the signature box are unacceptable.) *** Please delete as appropriate. Delete all of the bold if you were on the original authorship list and are remaining as an author.**

	First name	Family name		Signature	Date
~~1st~~ author 11	MICHELA	PACIFICO	I agree to the proposed new authorship shown in section 4 /~~and the addition/removal*of my name to the authorship list /and the proposed change in corresponding author~~		
~~2nd~~ author 12	ANGELICA	CROTTINI	I agree to the proposed new authorship shown in section 4 /~~and the addition/removal*of my name to the authorship list /and the proposed change in corresponding author~~		
~~3rd~~ author 13	ANDERS PAPE	MØLLER	I agree to the proposed new authorship shown in section 4 /~~and the addition/removal*of my name to the authorship list /and the proposed change in corresponding author~~		mm/dd/yyyy
~~4th~~ authors 14	RICHARD Z.	CHEN	I agree to the proposed new authorship shown in section 4 /and the addition/~~removal~~*of my name to the authorship list /~~and the proposed change in corresponding author~~	RZ Chen	11/17/2021
~~5th~~ author 15	THOMAS F.	VOGT	I agree to the proposed new authorship shown in section 4 /and the addition/~~removal~~*of my name to the authorship list /~~and the proposed change in corresponding author~~		
~~6th~~ author 16	GIULIO	PAVESI	I agree to the proposed new authorship shown in section 4 /~~and the addition/removal*of my name to the authorship list /and the proposed change in corresponding author~~		
~~7th~~ author 17	DAVID STEPHEN	HORNER	I agree to the proposed new authorship shown in section 4 /~~and the addition/removal*of my name to the authorship list /and the proposed change in corresponding author~~		

SPRINGER NATURE

Change of authorship request form - Journals (pre-acceptance)

Section 6: Declaration of agreement. *All* authors, unchanged, new and removed *must* sign this declaration.
(NB: Please print the form, (docu)-sign and return/upload a scanned copy. Please note that signatures that have been inserted as an image file are acceptable as long as it is handwritten. Typed names in the signature box are unacceptable.) *** Please delete as appropriate. Delete all of the bold if you were on the original authorship list and are remaining as an author.**

	First name	Family name		Signature	Date
11 author	MICHELA	PACIFICO	I agree to the proposed new authorship shown in section 4 /~~and the addition/removal*of my name to the authorship list /and the proposed change in corresponding author~~		
12 author	ANGELICA	CROTTINI	I agree to the proposed new authorship shown in section 4 /~~and the addition/removal*of my name to the authorship list /and the proposed change in corresponding author~~		
13 author	ANDERS PAPE	MØLLER	I agree to the proposed new authorship shown in section 4 /~~and the addition/removal*of my name to the authorship list /and the proposed change in corresponding author~~		mm/dd/yyyy
14 authors	RICHARD Z.	CHEN	I agree to the proposed new authorship shown in section 4 /and the addition/~~removal~~*of my name to the authorship list /~~and the proposed change in corresponding author~~	R Z Chen	11/17/202
15 author	THOMAS F.	VOGT	I agree to the proposed new authorship shown in section 4 /and the addition/~~removal~~*of my name to the authorship list /~~and the proposed change in corresponding author~~	Thomas F Vogt	mm/dd/yy 11/17/21
16 author	GIULIO	PAVESI	I agree to the proposed new authorship shown in section 4 /~~and the addition/removal*of my name to the authorship list /and the proposed change in corresponding author~~		
17 author	DAVID STEPHEN	HORNER	I agree to the proposed new authorship shown in section 4 /~~and the addition/removal*of my name to the authorship list /and the proposed change in corresponding author~~		

SPRINGER NATURE Springer Nature is one of the world's leading global research, educational and professional publishers, created in May 2015 through the combination of Nature Publishing Group, Palgrave Macmillan, Macmillan Education and Springer Science+Business Media.

SPRINGER NATURE

Change of authorship request form - Journals (pre-acceptance)

Section 6: Declaration of agreement. ***All*** **authors, unchanged, new and removed** ***must*** **sign this declaration.**
(NB: Please print the form, (docu)-sign and return/upload a scanned copy. Please note that signatures that have been inserted as an image file are acceptable as long as it is handwritten. Typed names in the signature box are unacceptable.) *** Please delete as appropriate. Delete all of the bold if you were on the original authorship list and are remaining as an author.**

	First name	Family name		Signature	Date
~~1st~~ author 11	MICHELA	PACIFICO	I agree to the proposed new authorship shown in section 4 /~~and the addition/removal*of my name to the authorship list /and the proposed change in corresponding author~~		
~~2nd~~ author 12	ANGELICA	CROTTINI	I agree to the proposed new authorship shown in section 4 /~~and the addition/removal*of my name to the authorship list /and the proposed change in corresponding author~~		
~~3rd~~ author 13	ANDERS PAPE	MØLLER	I agree to the proposed new authorship shown in section 4 /~~and the addition/removal*of my name to the authorship list /and the proposed change in corresponding author~~		
~~4th~~ authors 14	RICHARD Z.	CHEN	I agree to the proposed new authorship shown in section 4 /**and the addition/~~removal~~*of my name to the authorship list** /~~and the proposed change in corresponding author~~		
~~5th~~ author 15	THOMAS F.	VOGT	I agree to the proposed new authorship shown in section 4 /**and the addition/~~removal~~*of my name to the authorship list** /~~and the proposed change in corresponding author~~		
~~6th~~ author 16	GIULIO	PAVESI	I agree to the proposed new authorship shown in section 4 /~~and the addition/removal*of my name to the authorship list /and the proposed change in corresponding author~~	Giulio Pavesi	mm/dd/yyyy 11/11/2021
~~7th~~ author 17	DAVID STEPHEN	HORNER	I agree to the proposed new authorship shown in section 4 /~~and the addition/removal*of my name to the authorship list /and the proposed change in corresponding author~~		

SPRINGER NATURE Springer Nature is one of the world's leading global research, educational and professional publishers, created in May 2015 through the combination of Nature Publishing Group, Palgrave Macmillan, Macmillan Education and Springer Science+Business Media.

SPRINGER NATURE

Change of authorship request form - Journals (pre-acceptance)

Section 6: Declaration of agreement. ***All*** **authors, unchanged, new and removed** ***must*** **sign this declaration.**
(NB: Please print the form, (docu)-sign and return/upload a scanned copy. Please note that signatures that have been inserted as an image file are acceptable as long as it is handwritten. Typed names in the signature box are unacceptable.) *** Please delete as appropriate. Delete all of the bold if you were on the original authorship list and are remaining as an author.**

	First name	Family name		Signature	Date
~~1st~~ author<br>11	MICHELA	PACIFICO	I agree to the proposed new authorship shown in section 4 /~~and the addition/removal*of my name to the authorship list /and the proposed change in corresponding author~~		
~~2nd~~ author<br>12	ANGELICA	CROTTINI	I agree to the proposed new authorship shown in section 4 /~~and the addition/removal*of my name to the authorship list /and the proposed change in corresponding author~~		
~~3rd~~ author<br>13	ANDERS PAPE	MØLLER	I agree to the proposed new authorship shown in section 4 /~~and the addition/removal*of my name to the authorship list /and the proposed change in corresponding author~~		
~~4th~~ authors<br>14	RICHARD Z.	CHEN	I agree to the proposed new authorship shown in section 4 /**and the addition/~~removal~~*of my name to the authorship list** /~~and the proposed change in corresponding author~~		
~~5th~~ author<br>15	THOMAS F.	VOGT	I agree to the proposed new authorship shown in section 4 /**and the addition/~~removal~~*of my name to the authorship list** /~~and the proposed change in corresponding author~~		
~~6th~~ author<br>16	GIULIO	PAVESI	I agree to the proposed new authorship shown in section 4 /~~and the addition/removal*of my name to the authorship list /and the proposed change in corresponding author~~		
~~7th~~ author<br>17	DAVID STEPHEN	HORNER	I agree to the proposed new authorship shown in section 4 /~~and the addition/removal*of my name to the authorship list /and the proposed change in corresponding author~~	[signature]	mm/dd/yyyy<br>11/11/2021

SPRINGER NATURE Springer Nature is one of the world's leading global research, educational and professional publishers, created in May 2015 through the combination of Nature Publishing Group, Palgrave Macmillan, Macmillan Education and Springer Science+Business Media.

SPRINGER NATURE

Change of authorship request form - Journals (pre-acceptance)

	First name	Family name		Signature	Date
~~8th~~ author 18	NICOLA	SAINO	I agree to the proposed new authorship shown in section 4 ~~/and the addition/removal*of my name to the authorship list /and the proposed change in corresponding author~~	DECEASED	/
~~8th~~ author 19	ELENA	CATTANEO	I agree to the proposed new authorship shown in section 4 ~~/and the addition/removal*of my name to the authorship list /and the proposed change in corresponding author~~	Elena Cattaneo	mm/dd/yyyy 11/23/2021
~~10th author~~			I agree to the proposed new authorship shown in section 4 **/and the addition/removal*of my name to the authorship list** /and the proposed change in corresponding author		

Please use an additional sheet if there are more than 10 authors.

In case of author collaborations with formal agreement:

	Name of consortium/consortia	First name	Family name		Signature	Date
Representative/legal guarantor				I agree to the proposed new authorship shown in section 4 **/and the addition/removal*of my name to the authorship list** /and the proposed change in corresponding author		

Both added/removed authors should complete the information in the first table under Section 6.

---- End of form ----

SPRINGER NATURE Springer Nature is one of the world's leading global research, educational and professional publishers, created in May 2015 through the combination of Nature Publishing Group, Palgrave Macmillan, Macmillan Education and Springer Science+Business Media.
