# Supplementary material for: The evolutionary history of the polyQ tract in huntingtin sheds light on its functional pro-neural activities
Source: Cell Death Differ. 2022 Jan 1;29(2):293–305. doi: 10.1038/s41418-021-00914-9 (PMC8817008; doi:10.1038/s41418-021-00914-9)
Supplement: Supplementary file 29 — Dataset 22 [file 41418_2021_914_MOESM29_ESM.docx]

| **Group** | **Primer FW** | **Primer RV** |
| --- | --- | --- |
| Human | 5’-ttgctgtgtgaggcagaacc-3’ | 5’-gcagttaaaagaacccccgc-3’ |
| NHPs | 5’-tggctctgtgaggcagaaca-3’ | 5’-caacacagttaaacccccgc-3’ |
| Mammals | 5’-atggcgaccctggagaagctg-3’ | 5’-ggtcggtgcagcggctcct-3’ |
| Birds | 5’-atggccaccatggagaagctg-3’ | 5’-ggtctctggagcggctcct-3’ |
| Reptiles | 5’-atggccaccatggagaagctg-3’ | 5’-ggtctctggagcggctcct-3’ |
| Fishes | 5’-atggccaccatggagaaattg-3’ | 5’-atggccaccatggagaaattg-3’ |

Reaction protocol

Buffer HF 10x 1.5 μL

Mg_2_SO_4_ 50 mM 0.24 μL

dNTPs 10 mM 0.3 μL

Primer FW 10 uM 0.6 μL

Primer RV 10 uM 0.6 μL

Platinum Taq DNA Polymerase High Fidelity (Cat. no.11304-011) 0.15 μL

H_2_O to volume (15 μL)

Template DNA 3 μL

|  | Human | NHPs | Mammals | Birds | Reptiles | Fishes |
| --- | --- | --- | --- | --- | --- | --- |
| Initial denaturation | 10’ , 96 °C | 10’ , 96 °C | 10’ , 96 °C | 10’ , 96 °C | 10’ , 96 °C | 10’ , 96 °C |
| 36 cycles: |  | | | | | |
| Denaturation | 45’’, 96 °C | 45’’, 96 °C | 45’’, 96 °C | 45’’, 96 °C | 45’’, 96 °C | 45’’, 96 °C |
| Annealing | 45’’, 60 °C | 45’’, 60 °C | 45’’, 58 °C | 45’’, 58 °C | 45’’, 58 °C | 45’’, 55 °C |
| Extension | 1.5’, 68 °C | 1’, 68 °C | 1’, 68 °C | 1’, 68 °C | 1’, 68 °C | 30’’, 68 °C |
| Final extension | 10’, 68 °C | 10’, 68 °C | 10’, 68 °C | 10’, 68 °C | 10’, 68 °C | 10’, 68 °C |
